# Association of maternal lipid profile and gestational diabetes mellitus: A systematic review and meta-analysis of 292 studies and 97,880 women

**DOI:** 10.1016/j.eclinm.2021.100830

**Published:** 2021-04-16

**Authors:** Jiamiao Hu, Clare L. Gillies, Shaoling Lin, Zoe A. Stewart, Sarah E. Melford, Keith R. Abrams, Philip N. Baker, Kamlesh Khunti, Bee K. Tan

**Affiliations:** a College of Food Science, Fujian Agriculture and Forestry University, Fuzhou 350002, PR China; b Department of Cardiovascular Sciences and Diabetes Research Centre, University of Leicester, Leicester LE1 7RH, United Kingdom; c Diabetes Research Centre, Leicester General Hospital, Leicester LE5 4PW, United Kingdom; d Health Sciences, University of Leicester, Leicester LE1 7RH, United Kingdom

**Keywords:** Gestational diabetes mellitus, Lipids, Triglyceride, Very low-density lipoprotein cholesterol, Oral glucose tolerance test

## Abstract

**Background:**

Gestational Diabetes Mellitus (GDM) is the most prevalent metabolic disorder during pregnancy, however, the association between dyslipidaemia and GDM remains unclear.

**Methods:**

We searched Medline, Scopus, Web of Science, Cochrane, Maternity and Infant Care database (MIDIRS) and ClinicalTrials.gov up to February 2021 for relevant studies which reported on the circulating lipid profile during pregnancy, in women with and without GDM. Publications describing original data with at least one raw lipid [triglyceride (TG), total cholesterol (TC), high-density lipoprotein cholesterol (HDL-C), low-density lipoprotein cholesterol (LDL-C), or very low-density lipoprotein cholesterol (VLDL-C)] measurement were retained. Data extraction was performed using a piloted data extraction form. The protocol was registered with PROSPERO (CRD42019139696).

**Findings:**

A total of 292 studies, comprising of 97,880 pregnant women (28232 GDM and 69,648 controls) were included. Using random-effects meta-analysis models to pool study estimates, women with GDM had significantly higher (by 20%) TG levels, with a pooled weighted mean difference between GDM and non-GDM pregnancies of 0.388 mM (0.336, 0.439, *p* < 0.001). Further analyses revealed elevated TG levels occur in the first trimester and persist afterwards. Meta-regression analyses showed that differences in TG levels between women with GDM and healthy controls were significantly associated with age, BMI, study continent, OGTT procedure, and GDM diagnosis criteria.

**Interpretation:**

Elevated lipids, particularly, TG, are associated with GDM.

**Research in context**

**Evidence before this study**

Gestational Diabetes Mellitus (GDM) is characterized by impaired glucose metabolism which is first discovered, or begins, during pregnancy. Previous studies have suggested an association between disordered glucose and lipid metabolism in the development of GDM, although results have been inconsistent and only one previously published meta-analysis performed in January 2014 with 60 qualified studies summarized the association between dyslipidaemia with the occurrence of GDM. Data were collected by conducting a systematic search of articles of interest in Medline, Web of Science, Scopus, Cochrane; Maternity and Infant Care database (MIDIRS); and ClinicalTrials.gov up to February 2021. Search strategies for each database used the following terms: “Diabetes, Gestational” or “Gestational diabetes” or “Pregnancy Induced Diabetes” or “Pregnancy-induced Diabetes” or “GDM”; Keyword for lipids: “HDL” or “High Density Lipoprotein” or “LDL” or “Low Density Lipoprotein” or “VLDL” or “Very Low Density Lipoprotein” or “Triglycerides” or “Total Cholesterol” or “Dyslipid” or “Hyperlipid” or “Hypertriglycerid” or “Hypercholesterol”.

**Added value of this study**

Our meta-analysis comprises 292 studies (97,880 women), providing an up-to-date and comprehensive analysis of published literature on the relationship between maternal lipid levels and GDM – the most extensive on this topic to date.

**Implications of all the available evidence**

Elevated circulating TG levels were identified in pregnant women with GDM. In addition, increased TGs were observed before the traditional time for the GDM diagnosis by OGTT. Elevated lipids, particularly, TG, could predict GDM in early pregnancy.

Alt-text: Unlabelled box

## 1 Introduction

Gestational diabetes mellitus (GDM) is the most prevalent metabolic disorder during pregnancy affecting up to 25% of pregnancies in some countries [1]. Women with GDM face a higher risk of pre-eclampsia, macrosomia, pre-term birth, caesarean delivery, and stillbirth [2], [3], [4]. Infants of women with GDM have higher rates of neonatal hypoglycaemia and admission to neonatal intensive care units [4]. Further, women who develop GDM have approximately 10 times higher risk of T2DM later in life, and up to half will develop T2DM within 10 years after delivery [5,6]. Infants of mothers with GDM also face a higher risk of glucose intolerance, obesity, and diabetes during childhood and as adults.

The best screening program to detect GDM remains controversial [7]. Currently, the American Diabetes Association recommends pregnant women undertake GDM screening with the Oral Glucose Tolerance Test (OGTT) at 24 to 28 weeks of gestation [8]. However, universal screening for GDM using this strategy can pose challenges in rural or low-resource settings, where fasting or prolonged testing (as is required for the OGTT) is not practical, or costs are prohibitive. Further, screening at 24 to 28 weeks gestation may be too late to prevent some of the complications associated with GDM. Earlier screening for GDM could allow for more targeted testing and earlier intervention for at-risk groups, which in turn could mitigate adverse pregnancy outcomes.

Although the cause of GDM is not fully understood, maternal obesity, older maternal age, and women from certain ethnic groups have been identified as being at high risk [9]. Increasingly, attention has been given to the associations between impaired glucose metabolism, abnormal circulating lipid levels, and consequent worsening of glucose intolerance [10]. While the exact relationship between maternal plasma lipid metabolism and maternal glucose remain unclear, recent studies have highlighted that GDM induces a state of dyslipidaemia consistent with insulin resistance [11,12].

Although numerous data on this topic has been generated from clinical studies, a lack of a comprehensive and up-to-date meta-analysis makes it difficult for researchers to interpret the data in the existing literature. To the best of our knowledge, there is only one meta-analysis undertaken on this topic published on data up to 2014 [11].

## 2 Methods

The aim of this review was to identify studies that had reported circulating lipid levels in women with and without GDM, to assess if mean levels differed between the two groups. In addition, we also focused on whether changes in lipid profiles occur before and after the traditional OGTT diagnosis at 24 to 28 weeks of gestation.

### 2.1 Search strategy and selection criteria

The Systematic Reviews and Meta-Analyses (PRISMA) Guidelines were followed in this study [13]. The required data were collected by conducting a systematic search of articles of interest in Medline, Web of Science, Scopus, Cochrane; Maternity and Infant Care database (MIDIRS); and ClinicalTrials.gov up to February 2021.

Search strategies for each database were developed using the following terms: “Diabetes, Gestational” or “Gestational diabetes” or “Pregnancy Induced Diabetes” or “Pregnancy-induced Diabetes” or “GDM”; Keyword for lipids: “HDL” or “High Density Lipoprotein” or “LDL” or “Low Density Lipoprotein” or “VLDL” or “Very Low Density Lipoprotein” or “Triglycerides” or “Total Cholesterol” or “Dyslipid*” or “Hyperlipid*” or “Hypertriglycerid*” or “Hypercholesterol*”. We limited our search to articles written in English or Chinese but did not place any restrictions on publication date.

The protocol was registered with the International Prospective Register for Systematic Reviews (PROSPERO) database: registration number CRD42019139696 (Supplementary Document 1).

### 2.2 Selection criteria

The inclusion criteria were studies reporting blood lipid profile tests during pregnancy in women with and without GDM. We selected original research articles according to the following inclusion criteria: (1) longitudinal studies and cross-sectional studies; (2) data reported as mean values and standard deviation (SD) or standard error (SE)) on serum lipid parameters (triglycerides, total cholesterol, HDL-C, LDL-C, VLDL-C) during or before pregnancy, in pregnant women with and without GDM; (3) articles published in the English or Chinese languages. The exclusion criteria were: (1) other types of diabetes apart from GDM; (2) review articles and meeting abstracts without any relevant data; (3) reported associations without any retrievable data; (4) lipid profiles reported using non-parametric statistics such as median values. Search results were managed in Endnote. Two reviewers checked the search results to identify papers for inclusion and any differences resolved through discussion. Both reviewers were of similar experience level and expertise.

The data extraction from full text articles was performed using a data extraction form (Supplementary Table 1) by J.H, checked by S.L. and further approved by B.K.T. The following data were collected: study characteristics [author, year of publication, digital object identifier (DOI) or PMID/WOS if no DOI was assigned, study location]; participant characteristics [number of women in each group, age, pre-pregnancy body mass index (BMI), BMI during pregnancy, OGTT protocols and diagnostic criteria, systolic blood pressure, diastolic blood pressure, and gestational weeks at blood draw], and blood lipid profiles including TG; TC; HDL-C; LDL-C; VLDL-C (mean; SD/SE). The cholesterol and triglycerides measurement units were converted to mM using online tools (https://www.omnicalculator.com/health/cholesterol-units) and SEs were converted to SDs. Study location was coded according to their continents. Data extraction was carried out independently by two researchers, with their results checked against each other, and disagreements resolved through discussion. Both researchers were of similar experience level and expertise. The risk of bias of each study was assessed using the Newcastle-Ottawa scale as assessed by two authors (J.H. and S.L.), independently. We judged studies that received a score of nine or eight stars to be at low risk of bias, studies that scored seven or six stars to be at medium risk, and those that scored five or less to be at high risk. The references of articles included in the meta-analysis are listed in Supplementary Table 1.

### 2.3 Data Analysis

The results of the eligible studies were pooled and as study heterogeneity was statistically significant, an overall estimate of effect size was calculated using a random effects meta-analysis. The outcome measure of this meta-analysis was the weighted mean difference (WMD) and 95% confidence interval (95% CI) of TG, TC, HDL-C, LDL-C, and VLDL-C between women with and without GDM. Separate models were fitted for each lipid sub-type, by pregnancy trimester. A sensitivity analysis was also performed for each model based on English language articles only. Between study heterogeneity was assessed by the I^2^ statistic, which provides an estimate of the percentage of variability across studies that is due to heterogeneity rather than chance alone [14]. The extent of variation among the effects observed in different studies is calculated as Tau^2^ [15]. Publication bias was evaluated using the Begg's test. For studies with repeated measurements in different gestational weeks, the first measurement was used for the analysis (the earliest measurement in each trimester was included in the sub-group analysis performed based on pregnancy trimester). Given the expected heterogeneity of the eligible studies, meta-regression analyses were also performed to relate the primary outcome with the individual study design characteristics. All statistical analyses were performed using Stata 16 (StataCorp., College Station, TX, USA).

### 2.4 Role of the funding source

The funding sources had no role in the design and conduct of the study, analyses, and interpretation of the data. The corresponding author had full access to all the data in the study and had final responsibility for the decision to submit for publication.

## 3 Results

### 3.1 Characteristics of Included Studies

**Fig. 1:**
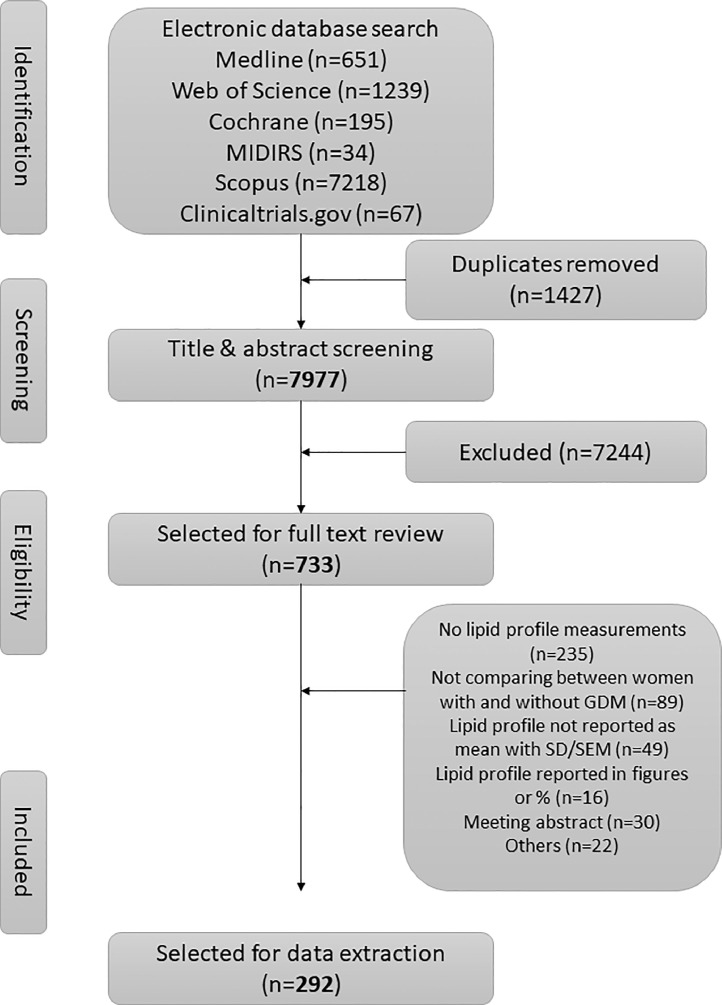
PRISMA flow chart of selection of studies.

Results from our search strategy are summarised in a PRISMA flowchart in Fig. 1. In total, we identified 9404 publications, 1427 were duplicates, and an additional 7244 were excluded based on reviewing the titles and abstracts. The remaining 733 articles underwent full-text review; 441 articles were additionally excluded. The most common reasons for exclusion after full-text review were studies not reporting circulating lipid profile measurements (*n* = 235), not comparing between women with and without GDM (*n* = 89) and lipid values reported using statistics other than the mean (*n* = 49). After final exclusions, 292 articles were eligible for inclusion in the meta-analyses.

Characteristics of the studies included in the meta-analyses are shown in Supplementary Table 1. Sample size ranged from 8 to 7773. Most of these studies were carried out in (*n* = 117), Turkey (*n* = 39), Spain (*n* = 19), Iran (*n* = 17) and USA (*n* = 14). Most of the included studies measured fasting lipid levels (89%). Maternal age measurements and BMI measurements were provided for most studies (88% and 84%, respectively; Supplementary Table 1).

Of the 292 included studies, 34 studies received nine out of nine stars for the Newcastle-Ottawa Score, 165 studies received eight stars, 86 studies received seven stars, and 7 studies received six stars (Supplementary Table 1)**.** The most poorly reported categories were cross sectional studies without a power calculation to justify the sample size.

### 3.2 Association between GDM and blood lipid profile

In this systematic review, we conducted WMD meta-analyses to compare two groups (women with and without GDM) for five outcomes (TG, TC, HDL-C, LDL-C, VLDL-C). Due to the large number of studies included, a forest plot was not appropriate to display the meta-analysis results, but instead results were summarized in Table 1 and Supplementary Tables 2–6. Women with GDM had significantly higher levels of TGs (WMD: 0.388 mM, 95% CI [0.336, 0.439], *p* < 0.001). In addition, significantly higher levels in total cholesterol (WMD: 0.149 mM, 95% CI [0.084, 0.214], *p* < 0.001), LDL-C (WMD: 0.079 mM, 95% CI [0.018, 0.140], *p* = 0.011) and VLDL-C (WMD: 0.216 mM, 95% CI [0.100, 0.332], *p* < 0.001) as well as significantly lower HDL-C levels (WMD: -0.079 mM, 95% CI [-0.100, -0.058], *p* < 0.001) were also observed. Since a physiological increase in plasma triglycerides and total cholesterol is a normal phenomenon as pregnancy progresses, expected Significant heterogeneities (I^2^ = 95.3%, 94.5%, 95.9%, 88.7% and 91.3%, respectively) were detected in all meta-analyses conducted using combined data from all trimesters. This is expected given the physiological increase in circulating TGs and TC as pregnancy progresses.

**Table 1:** Pooled weighted mean difference using random effects meta-analyses of circulating lipid levels between women with and without gestational diabetes mellitus.

Lipid		No. of studies	WMD (mM)	95% CI (mM)	*P*	I^2^ statistic	Tau^2^	Begg's *P*
**TG**		**258**	**0.388**	**0.336 to 0.439**	**<** **0.001**	**95.3%**	**0.1458**	**0.002**
	First trimester	44	0.239	0.187 to 0.291	< 0.001	84.4%	0.0224	0.863
	Second trimester	135	0.434	0.365 to 0.503	< 0.001	94.0%	0.1339	0.053
	Third trimester	95	0.376	0.255 to 0.498	< 0.001	96.5%	0.3035	0.010
	Fasting	227	0.403	0.345 to 0.461	< 0.001	95.6%	0.1659	0.072
	Excluding studies from China	161	0.328	0.280 to 0.377	< 0.001	88.0%	0.0659	0.161
**TC**		**261**	**0.149**	**0.084 to 0.214**	**<** **0.001**	**94.5%**	**0.2280**	**0.149**
	First trimester	47	0.137	0.079 to 0.196	< 0.001	84.3%	0.0274	0.854
	Second trimester	138	0.124	0.008 to 0.240	0.036	95.5%	0.4148	0.098
	Third trimester	90	0.037	-0.083 to 0.156	0.548	92.0%	0.2508	0.387
	Fasting	229	0.140	0.065 to 0.216	< 0.001	95.0%	0.2758	0.155
	Excluding studies from China	156	0.114	0.003 to 0.226	0.044	94.9%	0.4190	0.811
**HDL-C**		**232**	**-0.079**	**-0.100 to -0.058**	**<** **0.001**	**91.3%**	**0.0196**	**0.322**
	First trimester	40	-0.067	-0.093 to -0.041	< 0.001	82.9%	0.0048	0.484
	Second trimester	118	-0.069	-0.103 to -0.036	<0.001	93.5%	0.0277	0.329
	Third trimester	83	-0.104	-0.140 to -0.067	< 0.001	87.2%	0.0195	0.959
	Fasting	207	-0.078	-0.100 to -0.055	< 0.001	92.0%	0.0210	0.297
	Excluding studies from China	143	-0.072	-0.103 to -0.040	< 0.001	92.4%	0.0287	0.999
**LDL-C**		**228**	**0.079**	**0.018 to 0.140**	**0.011**	**95.9%**	**0.1810**	**0.041**
	First trimester	38	0.080	0.026 to 0.134	0.004	85.2%	0.0182	0.669
	Second trimester	119	0.048	-0.016 to 0.112	0.141	90.5%	0.0955	0.266
	Third trimester	79	-0.005	-0.182 to 0.171	0.953	97.8%	0.5604	0.000
	Fasting	203	0.078	0.010 to 0.145	0.024	96.2%	0.1969	0.029
	Excluding studies from China	137	0.065	0.003 to 0.127	0.041	85.4%	0.0902	0.404
**VLDL-C**		**17**	**0.216**	**0.100 to 0.332**	**<** **0.001**	**88.7%**	**0.0440**	**0.650**
	First trimester	3	0.240	-0.192 to 0.672	0.276	96.4%	0.1227	1.000
	Second trimester	9	0.194	0.146 to 0.243	< 0.001	0.0%	0.0000	0.917
	Third trimester	7	0.159	-0.139 to 0.458	0.296	92.3%	0.1271	0.764
	Fasting	15	0.238	0.115 to 0.360	< 0.001	88.7%	0.0439	0.767
	Excluding studies from China	15	0.222	0.095 to 0.348	0.001	89.9%	0.0488	0.921

Next, we stratified the time of lipid measurements according to trimesters. We found significantly higher TG levels in women with GDM compared to women without GDM for first trimester (WMD: 0.239 mM, 95% CI [0.187, 0.291]), second trimester (WMD: 0.434 mM, 95% CI [0.365, 0.503]) and third trimester (WMD: 0.376 mM, 95% CI [0.255, 0.498]) [Table 1]. The data for TC, HDL-C and LDL-C in women with GDM compared to women without GDM were similar (Table 1). VLDL-C levels between women with and without GDM appeared to fall as pregnancy duration increased, however, study numbers were limited and CIs very wide (Table 1).

### 3.3 Association between imbalance of age, BMI, blood pressure standards and outcomes

Meta-regression analyses showed a significant association with differences in mean TG and TC levels between women with GDM compared to women without GDM for BMI and blood pressure; no significant associations were found for pre-pregnancy BMI and gestational age (Table 2).

**Table 2:** Meta-regression analysis to predict the influence of differences of age, BMI, and blood pressure between women with and without gestational diabetes mellitus on lipid profile measurements.

Lipid	Covariate	No. of studies	Coef.	95% CI	*P*	I^2^_res
TG	Age difference	229	0.002	-0.002 to 0.005	0.312	94.59%
	Pre-BMI difference	118	0.017	-0.010 to 0.043	0.226	92.82%
	BMI difference	138	0.058	0.022 to 0.094	0.002	91.52%
	SBP difference	68	0.059	0.030 to 0.088	< 0.001	89.97%
	DBP difference	67	0.000	-0.001 to 0.001	0.927	92.39%
	Gestational weeks at blood sampling	117	0.003	-0.008 to 0.014	0.545	91.89%
TC	Age difference	231	0.000	-0.005 to 0.004	0.810	89.63%
	Pre-BMI difference	119	-0.010	-0.057 to 0.038	0.685	90.56%
	BMI difference	140	0.051	0.004 to 0.098	0.034	89.38%
	SBP difference	71	0.041	0.006 to 0.077	0.024	89.71%
	DBP difference	71	0.001	-0.001 to 0.002	0.486	91.19%
	Gestational weeks at blood sampling	120	-0.007	-0.020 to 0.007	0.344	96.04%
HDL-C	Age difference	209	0.001	-0.002 to 0.003	0.662	91.46%
	Pre-BMI difference	104	0.001	-0.021 to 0.022	0.936	83.05%
	BMI difference	134	-0.019	-0.044 to 0.005	0.114	92.54%
	SBP difference	65	-0.026	-0.050 to -0.002	0.033	93.80%
	DBP difference	64	0.000	0.000 to 0.000	0.900	80.20%
	Gestational weeks at blood sampling	108	-0.001	-0.009 to 0.006	0.743	93.02%
LDL-C	Age difference	206	0.001	-0.002 to 0.004	0.696	96.03%
	Pre-BMI difference	100	0.005	-0.050 to 0.060	0.856	91.86%
	BMI difference	130	0.030	-0.012 to 0.072	0.154	88.77%
	SBP difference	61	0.019	-0.015 to 0.053	0.266	89.74%
	DBP difference	61	0.000	-0.001 to 0.001	0.951	92.51%
	Gestational weeks at blood sampling	104	-0.005	-0.016 to 0.005	0.294	90.87%
VLDL-C	Age difference	16	0.002	-0.107 to 0.111	0.970	90.11%
	Pre-BMI difference	6	0.103	-0.319 to 0.526	0.535	93.50%
	BMI difference	11	0.089	-0.067 to 0.245	0.230	81.51%
	SBP difference	insufficient observations				
	DBP difference	insufficient observations				
	Gestational weeks at blood sampling	7	0.001	-0.077 to 0.080	0.970	79.36%

Note: Age/Pre-BMI/BMI/SBP/DBP differences were calculated as age/BMI before pregnancy/BMI during pregnancy/Systolic blood pressure /Distal blood pressure in women with GDM deducted by those parameters in pregnant women without GDM.

### 3.4 Association between continent of study and outcomes

**Fig. 2:**
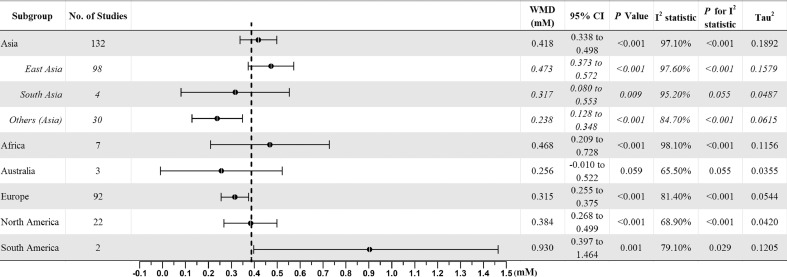
Forest plot of comparison: Circulating triglyceride levels in women with gestational diabetes mellitus versus control subjects (mM).

We found significantly higher TG levels in women with GDM compared to women without GDM (Table 3). Studies conducted in South America showed the largest pooled difference in TG levels compared to other continents (WMD: 0.930 mM, 95% [0.397, 1.464], *p* < 0.001), however, study numbers were low, with only two studies from Brazil (Table 3). When we subdivided studies conducted in Asia into East Asia (China and Japan), South Asia (India and Pakistan) and other Asian countries, we found that East Asians showed a higher WMD in TG levels (WMD: 0.473mM, 95% [0.373, 0.572]) compared to Europeans (WMD 0.315mM, 95% CI [0.255, 0.375]) whilst South Asians and other Asians had lower WMDs in TG levels (Fig. 2).

**Table 3:** Subgroup analysis of circulating lipid levels between women with and without gestational diabetes mellitus stratified by locations of studies.

Lipid	Continents	No. of studies	WMD (mM)	95% CI (mM)	*P*	I^2^ statistic	*P* for I^2^ statistic	Tau^2^
TG	Asia	132	0.418	0.338 to 0.498	< 0.001	97.1%	< 0.001	0.1892
	Africa	7	0.468	0.209 to 0.728	< 0.001	98.1%	< 0.001	0.1156
	Australia	3	0.256	-0.010 to 0.522	0.059	65.5%	0.055	0.0355
	Europe	92	0.315	0.255 to 0.375	< 0.001	81.4%	< 0.001	0.0544
	North America	22	0.384	0.268 to 0.499	< 0.001	68.9%	< 0.001	0.0420
	South America	2	0.930	0.397 to 1.464	0.001	79.1%	0.029	0.1205
TC	Asia	140	0.191	0.101 to 0.280	< 0.001	96.2%	< 0.001	0.2472
	Africa	7	0.180	-0.301 to 0.661	0.464	98.1%	< 0.001	0.3911
	Australia	1	-0.775	-1.742 to 0.191	0.116	–	–	0.0000
	Europe	90	0.120	0.032 to 0.209	0.008	81.7%	< 0.001	0.1186
	North America	19	-0.070	-0.198 to 0.059	0.289	35.8%	0.061	0.0246
	South America	4	0.660	0.275 to 1.045	0.001	81.6%	0.001	0.1167
HDL-C	Asia	121	-0.083	-0.107 to -0.059	< 0.001	90.3%	< 0.001	0.0133
	Africa	5	-0.122	-0.222 to -0.022	0.017	75.4%	0.003	0.0081
	Australia	1	2.080	1.927 to 2.233	< 0.001	–	–	0.0000
	Europe	88	-0.100	-0.132 to -0.068	< 0.001	82.3%	< 0.001	0.0140
	North America	13	-0.086	-0.142 to -0.030	0.003	63.3%	0.001	0.0056
	South America	4	0.046	-0.149 to 0.242	0.641	93.3%	< 0.001	0.0345
LDL-C	Asia	122	0.096	0.013 to 0.179	0.024	97.4%	< 0.001	0.1909
	Africa	5	0.435	-0.226 to 1.095	0.197	97.5%	< 0.001	0.5238
	Australia	0		–	–	–	–	–
	Europe	84	0.069	-0.018 to 0.156	0.120	85.2%	< 0.001	0.1066
	North America	13	-0.173	-0.292 to -0.054	0.004	46.2%	0.034	0.0186
	South America	4	0.199	-0.048 to 0.445	0.115	55.8%	0.079	0.0318
VLDL-C	Asia	5	0.168	0.101 to 0.234	< 0.001	0.0%	0.767	0.0000
	Africa	1	0.552	0.447 to 0.658	< 0.00	–	–	0.0000
	Australia	0	–	–	–	–	–	–
	Europe	9	0.265	0.091 to 0.439	0.003	89.6%	< 0.001	0.0546
	North America	2	-0.206	-0.764 to 0.352	0.469	71.5%	0.061	0.1249
	South America	0	–	–	–	–	–	–

### 3.5 Association between OGTT procedures/diagnostic standards and outcomes

Studies in this meta-analysis adopted various OGTT procedures and diagnostic standards as summarized in Table 4 and Supplementary Table 7. Subgroup analyses showed that the mean difference in TG levels between women with GDM compared to women without GDM was greater in studies that adopted a 100 g, 3 h OGTT compared to studies using the 75 g, 2 h OGTT (Table 4 and Supplementary Table 7); this may be partially explained by further subgroup analyses stratifying by diagnostic standards (Table 4). The differences in TG levels between women with GDM compared to women without GDM were more than 0.373 mM in studies that used the 100 g, 3 h OGTT, whilst studies using the 75 g, 2 h OGTT observed TG differences less than 0.375 mM. However, no differences were found for other lipids (Supplementary Table 7).

**Table 4:** Subgroup analysis of circulating TG levels between women with and without gestational diabetes mellitus stratified by OGTT procedures and diagnostic standards.

OGTT	No. of studies	WMD (mM)	95% CI (mM)	*p*-value	I^2^ statistic	*p*-value	Tau^2^
75 g, 2 h OGTT	137	0.309	0.242 to 0.375	< 0.001	95.1%	< 0.001	0.1295
5.3/10.0/8.6	10	0.258	0.065 to 0.451	0.009	90.0%	< 0.001	0.0817
5.5/-/8.0	4	0.199	-0.092 to 0.491	0.180	77.3%	0.004	0.0638
6.0/-/9.0	1	0.020	-0.035 to 0.075	0.480	–	–	0.0000
5.1/10.0/8.5	102	0.318	0.236 to 0.399	< 0.001	95.8%	< 0.001	0.1485
7.0(6.1)/-/7.8	20	0.297	0.184 to 0.410	< 0.001	76.5%	< 0.001	0.0384
100 g, 3 h OGTT	76	0.459	0.373 to 0.545	< 0.001	90.2%	< 0.001	0.1089
5.3/10.0/8.6/7.8	47	0.428	0.337 to 0.519	< 0.001	87.8%	< 0.001	0.0719
5.8/10.6/9.2/8.0	26	0.522	0.310 to 0.734	< 0.001	93.2%	< 0.001	0.2525
5.0/9.2/8.0/7.0	3	0.828	0.104 to 1.552	0.025	73.4%	0.023	0.2944

## 4 Discussion

We show that women with GDM have significantly higher circulating TGs (0.388 mM), TC (0.149 mM), LDL-C (0.079 mM), VLDL-C (0.216 mM) and lower HDL-C (-0.079 mM) compared to women without GDM. Among these outcomes, TGs was the most significant, with most of the included studies reporting higher TG levels in women with GDM. Furthermore, higher TG levels were found throughout pregnancy, in keeping with a previous meta-analysis on this topic published on data up to 2014 [11].

In addition, the mean differences in TG levels were ~0.4 mM. Considering that individuals usually have TG levels of 1.69 mM or below, this equates to an approximately 20–25% increase in TG levels in women with GDM (the average TG level in women with GDM was 2.55 mM). Notably, higher TG levels were observed in the first trimester of pregnancy, and thus could potentially be integrated into a risk stratification algorithm to calculate the risk of GDM allowing for early intervention to mitigate adverse pregnancy outcomes.

Compared to the previous meta-analysis [11], our meta-analysis was greatly strengthened by adopting a comprehensive strategy and inclusion criteria, which yielded a high number of eligible publications (60 publications in the previous meta-analysis, 292 publications in our meta-analysis). This meta-analysis also included both Chinese and English language publications (in different populations in different continents), allowing a broader range of included studies. All studies included in this meta-analysis received a score > 5 for the Newcastle-Ottawa quality assessment, indicating none were at high risk of bias. We also conducted full-text analyses of manuscripts even when no lipid-related information was available in the title and abstract. Therefore, our meta-analysis included studies that had reported lipid levels only as background data allowing the inclusion of a number of studies reporting no significant differences in lipids between women with and without GDM; this lowers the impact of publication bias. Additionally, our meta-analysis also revealed several novel findings. Firstly, the elevation of TG levels in women with GDM seems to be more pronounced in certain populations i.e. South Americans and East Asians, which may suggest more severe complications associated with GDM in these populations. Secondly, differences in diagnostic and screening protocols were also associated with variations in TG level differences between women with GDM compared to women without GDM. Thus, our findings have significant implications for many more populations compared to the previous meta-analysis [11].

High heterogeneity was observed in our study, suggesting TG levels could be influenced by several variables, including ethnicity and BMI, all of which have been identified as risk factors for GDM. Notably, there is significant heterogeneity by continent and although there were only two studies from South America, the results were quite different compared to other populations. Furthermore, the heterogeneity could reflect metabolic heterogeneity resulting in different degrees of insulin resistance in women with and without GDM. Future investigation is needed to explore the underlying mechanisms driving these differences. In addition, our analyses also showed that blood pressure differences were associated with TG levels, consistent with observations outside pregnancy in conditions such as type 2 diabetes [16].

There is now convincing evidence of biological plausibility linking hyperglycaemia and dyslipidaemia. For women with GDM, insulin resistance and higher oestrogen levels during pregnancy may cause the normal pregnancy-associated fluctuation of lipid metabolism to exceed the physiological adaptation. The consequences of this hyperglycaemia and dyslipidaemia on the developing fetus include endothelial dysfunction of the fetoplacental vasculature and the development of fetal aortic atherosclerosis [12], which predisposes children born to mothers with GDM to the development of cardiovascular disease later in their adulthood. Furthermore, a multiple-mediator path model using robust maximum likelihood estimation demonstrated that increasing maternal insulin resistance and glucose levels, even within normal range, are responsible for 21% of the association between pre-pregnancy BMI and neonatal adiposity; insulin resistance was also found to be positively associated with maternal TG levels [17].

A limitation of our meta-analysis pertains to sub-group analysis on fetal sex effects. The risk of GDM and associated outcomes differ between women bearing a male compared to a female fetus [18]. Also, it would be interesting to establish a dose-response relationship between lipids and GDM. However, we did not have individual patient data to perform either of these analyses.

In summary, this is the largest and most up to date meta-analysis aimed at investigating the association between dyslipidaemia and GDM. The findings from this study also suggest that elevated lipid levels, particularly TGs, is associated with future risk of GDM, and could potentially be integrated into a risk stratification algorithm to calculate the risk of GDM.

## Author contributions

Data extraction was done by JH and SL and the analyses were done by JH, SL and replicated by CG, supervised by KK and BKT. BKT conceived the idea for this project and acquired funding for this study. All authors were involved in data interpretation and the writing or editing of the report.

## Funding

This study was supported by University of Leicester Global Challenge Research Fund Visiting Fellowships; 10.13039/501100001809Natural Science Foundation of China (81703065); the National Institute for Health Research for Applied Research Collaboration – East Midlands (NIHR ARC-EM); Leicester Biomedical Research Centre (BRC) and Medical Research Council (MRC), UK.

## Data sharing statement

All data used and generated in this study is available in the manuscript and supporting information.

## Declaration of Competing Interest

KK reports personal fees from Amgen, AstraZeneca, Bayer, NAPP, Lilly, Merck Sharp & Dohme, Novartis, Novo Nordisk, Roche, Berlin-Chemie AG / Menarini Group, Boehringer-Ingelheim, Sanofi-Aventis and Servier, other from Astrazeneca, Lilly, Merck Sharp & Dohme, Novo Nordisk, Sanofi-Aventis, grants from AstraZeneca, Novartis, Novo Nordisk, Sanofi-Aventis, Lilly, Servier, Pfizer, Boehringer Ingelheim and Merck Sharp & Dohme, outside the submitted work. KA reports grants from BHF, HDR UK, MRC and National Institute for Health Research during the course of the study. KA is a member of the NICE Decision Support Unit (DSU) and the NICE Technical Support Unit (TSU) and has provided strategic and methodological advice to NICE. KA is a standing member of the NICE Diagnostics Advisory Committee (DAC) and a member of the Health Data Research (HDR) UK/UKRI/SAGE COVID-19 Task Force. JH, CG, SL, ZS, SM, PB and BKT have no competing interests to declare.

## Appendix Supplementary materials

Image, application 1

Image, application 2

Image, application 3

Image, application 4

Image, application 5

Image, application 6

Image, application 7

Image, application 8
