# Supplementary material for: Association of maternal lipid profile and gestational diabetes mellitus: A systematic review and meta-analysis of 292 studies and 97,880 women
Source: eClinicalMedicine. 2021 Apr 16;34:100830. doi: 10.1016/j.eclinm.2021.100830 (PMC8102708; doi:10.1016/j.eclinm.2021.100830)
Supplement: Supplementary file 2 [file mmc2.pdf]

# Systematic review

To edit the record click Start an update below. This will create a new version of the record - the existing version will remain unchanged.

## 1. * Review title.

Give the title of the review in English

A systematic review to investigate circulating lipid levels in pregnant women with and without gestational diabetes mellitus

## 2. Original language title.

For reviews in languages other than English, give the title in the original language. This will be displayed with the English language title.

## 3. * Anticipated or actual start date.

Give the date the systematic review started or is expected to start.

29/04/2019

## 4. * Anticipated completion date.

Give the date by which the review is expected to be completed.

20/03/2021

## 5. * Stage of review at time of this submission.

Tick the boxes to show which review tasks have been started and which have been completed.
Update this field each time any amendments are made to a published record.

**Reviews that have started data extraction (at the time of initial submission) are not eligible for inclusion in PROSPERO**.
If there is later evidence that incorrect status and/or completion date has been supplied, the published PROSPERO record will be marked as retracted.

This field uses answers to initial screening questions. It cannot be edited until after registration.

The review has not yet started: No

| Review stage | Started | Completed |
|---|---|---|
| Preliminary searches | Yes | No |
| Piloting of the study selection process | No | No |
| Formal screening of search results against eligibility criteria | No | No |
| Data extraction | No | No |
| Risk of bias (quality) assessment | No | No |
| Data analysis | No | No |

Provide any other relevant information about the stage of the review here.

## 6. * Named contact.

The named contact is the guarantor for the accuracy of the information in the register record. This may be any member of the review team.

Jiamiao Hu

Email salutation (e.g. "Dr Smith" or "Joanne") for correspondence:
Dr Hu

## 7. * Named contact email.

Give the electronic email address of the named contact.

jiamiao.hu@hotmail.com

## 8. Named contact address

**PLEASE NOTE this information will be published in the PROSPERO record so please do not enter private information, i.e. personal home address**

Give the full institutional/organisational postal address for the named contact.

Leicester Institute for Advanced Studies

## 9. Named contact phone number.

Give the telephone number for the named contact, including international dialling code.

+86-15060681086

## 10. * Organisational affiliation of the review.

Full title of the organisational affiliations for this review and website address if available. This field may be completed as 'None' if the review is not affiliated to any organisation.

Leicester Institute for Advanced Studies

Organisation web address:
https://www2.le.ac.uk/institution/lias

## 11. * Review team members and their organisational affiliations.

Give the personal details and the organisational affiliations of each member of the review team. Affiliation refers to groups or organisations to which review team members belong.
**NOTE: email and country now MUST be entered for each person, unless you are amending a published record.**

Dr Jiamiao Hu. Leicester Institute for Advanced Studies, University of Leicester
Dr Clare Gillies. University of Leicester
Professor Bee Kang Tan. University of Leicester

## 12. * Funding sources/sponsors.

Details of the individuals, organizations, groups, companies or other legal entities who have funded or sponsored the review.

GCRF Fellowship

Grant number(s)

State the funder, grant or award number and the date of award

## 13. * Conflicts of interest.

List actual or perceived conflicts of interest (financial or academic).

None

## 14. Collaborators.

Give the name and affiliation of any individuals or organisations who are working on the review but who are not listed as review team members. **NOTE: email and country must be completed for each person, unless you are amending a published record.**

Shaoling Lin. Fujian Agriculture and Forestry University

## 15. * Review question.

State the review question(s) clearly and precisely. It may be appropriate to break very broad questions down into a series of related more specific questions. Questions may be framed or refined using PI(E)COS or similar where relevant.

Do women with Gestational diabetes mellitus have higher lipid levels?

## 16. * Searches.

State the sources that will be searched (e.g. Medline). Give the search dates, and any restrictions (e.g. language or publication date). Do NOT enter the full search strategy (it may be provided as a link or attachment below.)

We conducted a systematic search of articles of interest in MEDLINE, Web of Science, Scopus, Cochrane Library and Maternity & Infant Care Database.

Search strategy
Search terms related to studies were combined in the following strategy
MeSH terms: "Gestational diabetes mellitus [MeSH]" and "Lipids"

Keywords for Gestational diabetes mellitus: "Gestational Diabetes" or "Diabetes Mellitus Gestational" or "Pregnancy-Induced Diabetes"

Keyword for lipids: "HDL" or "High Density Lipoprotein" or "LDL" or "Low Density Lipoprotein" or "VLDL" or "Very Low Density Lipoprotein" or "Triglycerides" or "Total Cholesterol" or "Dyslipidemia" or "Hyperlipidemia" or "Hypertriglyceridemia" or "Hypercholesterolemia" or "Lipids" .

We performed the search using above strategy in the database "MEDLINE", "Web of Science", "Cochrane Library", "Maternity & Infant Care Database (MIDIRS) & "Scopus" on 15/Mar/2021.

In the search syntax we did not added any restriction on publication dates, while we only selected articles written in English or Chinese.

## 17. URL to search strategy.

Upload a file with your search strategy, or an example of a search strategy for a specific database, (including the keywords) in pdf or word format. In doing so you are consenting to the file being made publicly accessible.

Or provide a URL or link to the strategy. Do NOT provide links to your search **results**.

Do not make this file publicly available until the review is complete

## 18. * Condition or domain being studied.

Give a short description of the disease, condition or healthcare domain being studied in your systematic review.

Gestational diabetes mellitus

## 19. * Participants/population.

Specify the participants or populations being studied in the review. The preferred format includes details of both inclusion and exclusion criteria.

Pregnant women with gestational diabetes mellitus (as diagnosed using any recognised diagnostic criteria)

## 20. * Intervention(s), exposure(s).

Give full and clear descriptions or definitions of the interventions or the exposures to be reviewed. The preferred format includes details of both inclusion and exclusion criteria.

Gestational diabetes mellitus.

## 21. * Comparator(s)/control.

Where relevant, give details of the alternatives against which the intervention/exposure will be compared (e.g. another intervention or a non-exposed control group). The preferred format includes details of both inclusion and exclusion criteria.

Pregnant women without gestational diabetes mellitus.

## 22. * Types of study to be included.

Give details of the study designs (e.g. RCT) that are eligible for inclusion in the review. The preferred format includes both inclusion and exclusion criteria. If there are no restrictions on the types of study, this should be stated.

Longitudinal studies and cross-sectional studies (cohort and case-control studies).

## 23. Context.

Give summary details of the setting or other relevant characteristics, which help define the inclusion or exclusion criteria.

## 24. * Main outcome(s).

Give the pre-specified main (most important) outcomes of the review, including details of how the outcome is defined and measured and when these measurement are made, if these are part of the review inclusion criteria.

The circulating lipid levels between women with and without gestational diabetes mellitus.

* Measures of effect
Not applicable.

## 25. * Additional outcome(s).

List the pre-specified additional outcomes of the review, with a similar level of detail to that required for main outcomes. Where there are no additional outcomes please state ‘None’ or ‘Not applicable’ as appropriate to the review

None.

* Measures of effect
Not applicable.

## 26. * Data extraction (selection and coding).

Describe how studies will be selected for inclusion. State what data will be extracted or obtained. State how this will be done and recorded.

All abstracts identified by the search strategy will be imported into Endnote to facilate the article selection process. Duplicates will be removed and two authors (J.H and S.L) will independently assess title and abstracts against inclusion criteria. Full text articles will then be screened for all articles that appear eligible. The two authors (J.H and C.G) will meet to discuss any disagreements, and a third author (B.K.T) will be consulted if

required. Data will be extracted independently by two authors (J.H and S.L) using a standardised data extraction form created for this review. Again, any disagreements will be resolved through discussion, involving third and fourth authors (C.G and B.K.T) where necessary. Data will be extracted on study details, patient characteristics and outcomes of interest, including the following data being collected from each included study: 1) Age; 2) BMI; 3) SBP; 4) DBP; 5) TG; 6) TC; 7) HDL-C; 8) LDL-C; 9) VLDL-C; 10) timing of blood sampling for lipid profile measurement; 11) OTGG method and diagnosis criteria ; 11) Author; 12) Year; 13) DOI; 14) Country of the study; 15) Ethnicity; Data will be imported into Stata for the analyses, and will be coded numerically wherever possible.

## 27. * Risk of bias (quality) assessment.

State which characteristics of the studies will be assessed and/or any formal risk of bias/quality assessment tools that will be used.

Risk of bias will be assessed using the Newcastle-Ottawa score. All selected studies are used for synthesis.

## 28. * Strategy for data synthesis.

Describe the methods you plan to use to synthesise data. This **must not be generic text** but should be **specific to your review** and describe how the proposed approach will be applied to your data.
If meta-analysis is planned, describe the models to be used, methods to explore statistical heterogeneity, and software package to be used.

The data extraction will be carried out by two individuals (J.H and S.L) and discrepancies will be resolved through discussion with a third reviewer (C.G or B.K.T). The data analysis will be carried out by one reviewer (J.H) and checked by C.G.

Aggregate participant data will be used in a quantitative synthesis of the study results, where a minimum of 10 studies report the required data. Random effects meta-analyses will be run (using the Stata command metan) to estimate the pooled weighted mean differences of triglycerides, total Cholesterol, HDL-C, LDL-C, and VLDL-C between pregnant women with and without gestational diabetes mellitus.

Meta-regression and sub-group analyses will be used to explore the impact on the effect size of between study heterogeneity. This will include the impact of timing of blood sampling for the lipid profile measurement, country of the study, ethnicity, mean study age, and publication date.

Publication bias will be assessed using Begg's and Egger's tests.

All analyses will be carried out in Stata 15.

## 29. * Analysis of subgroups or subsets.

State any planned investigation of ‘subgroups’. Be clear and specific about which type of study or participant will be included in each group or covariate investigated. State the planned analytic approach.

Subgroup analysis will be performed if the primary analysis indicates a significant difference between control and GDM women. The subgroup analyses will be done for subsets of participants (such as different gestational weeks, BMIs), or for subsets of studies (such as different geographical locations, years).

## 30. * Type and method of review.

Select the type of review, review method and health area from the lists below.

### Type of review

| | |
|---|---|
| Cost effectiveness | No |
| Diagnostic | No |
| Epidemiologic | No |
| Individual patient data (IPD) meta-analysis | No |
| Intervention | No |
| Meta-analysis | Yes |

| | |
|---|---|
| Methodology | No |
| Narrative synthesis | No |
| Network meta-analysis | No |
| Pre-clinical | No |
| Prevention | No |
| Prognostic | No |
| Prospective meta-analysis (PMA) | No |
| Review of reviews | No |
| Service delivery | No |
| Synthesis of qualitative studies | No |
| Systematic review | Yes |
| Other | No |

Health area of the review

| | |
|---|---|
| Alcohol/substance misuse/abuse | No |
| Blood and immune system | No |
| Cancer | No |
| Cardiovascular | No |
| Care of the elderly | No |
| Child health | No |
| Complementary therapies | No |
| COVID-19 | No |
| Crime and justice | No |
| Dental | No |
| Digestive system | No |
| Ear, nose and throat | No |
| Education | No |
| Endocrine and metabolic disorders | No |
| Eye disorders | No |
| General interest | No |

| | |
|---|---|
| Genetics | No |
| Health inequalities/health equity | No |
| Infections and infestations | No |
| International development | No |
| Mental health and behavioural conditions | No |
| Musculoskeletal | No |
| Neurological | No |
| Nursing | No |
| Obstetrics and gynaecology | Yes |
| Oral health | No |
| Palliative care | No |
| Perioperative care | No |
| Physiotherapy | No |
| Pregnancy and childbirth | Yes |
| Public health (including social determinants of health) | No |
| Rehabilitation | No |
| Respiratory disorders | No |
| Service delivery | No |
| Skin disorders | No |
| Social care | No |
| Surgery | No |
| Tropical Medicine | No |
| Urological | No |
| Wounds, injuries and accidents | No |
| Violence and abuse | No |

## 31. Language.

Select each language individually to add it to the list below, use the bin icon to remove any added in error.

Chinese-Simplified
English

There is an English language summary.

## 32. * Country.

Select the country in which the review is being carried out. For multi-national collaborations select all the countries involved.

England

## 33. Other registration details.

Name any other organisation where the systematic review title or protocol is registered (e.g. Campbell, or The Joanna Briggs Institute) together with any unique identification number assigned by them.
If extracted data will be stored and made available through a repository such as the Systematic Review Data Repository (SRDR), details and a link should be included here. If none, leave blank.

## 34. Reference and/or URL for published protocol.

If the protocol for this review is published provide details (authors, title and journal details, preferably in Vancouver format)

No I do not make this file publicly available until the review is complete

## 35. Dissemination plans.

Do you intend to publish the review on completion?

Yes

## 36. Keywords.

Give words or phrases that best describe the review. Separate keywords with a semicolon or new line. Keywords help PROSPERO users find your review (keywords do not appear in the public record but are included in searches). Be as specific and precise as possible. Avoid acronyms and abbreviations unless these are in wide use.

Gestational diabetes; lipids; pregnancy

## 37. Details of any existing review of the same topic by the same authors.

If you are registering an update of an existing review give details of the earlier versions and include a full bibliographic reference, if available.

## 38. * Current review status.

Update review status when the review is completed and when it is published.
New registrations must be ongoing so this field is not editable for initial submission.

Review_Ongoing

## 39. Any additional information.

Provide any other information relevant to the registration of this review.

## 40. Details of final report/publication(s) or preprints if available.

Leave empty until publication details are available OR you have a link to a preprint (NOTE: this field is not editable for initial submission).
List authors, title and journal details preferably in Vancouver format.
