# Supplementary material for: Association of maternal lipid profile and gestational diabetes mellitus: A systematic review and meta-analysis of 292 studies and 97,880 women
Source: eClinicalMedicine. 2021 Apr 16;34:100830. doi: 10.1016/j.eclinm.2021.100830 (PMC8102708; doi:10.1016/j.eclinm.2021.100830)
Supplement: Supplementary file 3 [file mmc3.docx]

Supplementary Table 2 Summary Weighted Mean Differences of TG from Meta-Analyses

-------------------------------------------------------------------------------

Author (Year) | Effect [95% Conf. Interval] % Weight

--------------------------------+----------------------------------------------

Abo-Elmatty, D. M.,et al (2019) | 0.568 0.442 0.695 0.46

Ademoglu, E., et al (2015) | 0.530 0.167 0.892 0.39

Akdeniz, F. T.,et al (2017) | 0.744 0.368 1.121 0.38

Akturk, M.,et al (2010) | 0.474 0.025 0.923 0.35

Akturk, M.,et al (2008) | 0.475 -0.061 1.012 0.32

Al-Ajlan.A.,et al (2018) | 0.200 0.072 0.328 0.46

Al-Daghri,N.,et al (2018) | 0.200 -0.018 0.418 0.44

Al-Hakeem, M.M., et al (2014) | 0.600 0.327 0.873 0.42

Al-Hakeem, M.M., et al. (2014) | 0.600 -3.417 4.617 0.02

Al-Rubeaan,K.,et al (2014) | 0.030 -0.136 0.196 0.45

Al-Saleh, E., et al. (2007) | -0.034 -0.391 0.323 0.39

Alanbay, I.,et al (2012) | 0.736 0.387 1.085 0.39

Alattas, O.S., et al (1995) | 1.000 -1.479 3.479 0.04

Altinova,A.,et al (2007) | 0.187 -0.230 0.605 0.36

Anjum,F.,et al (2019) | 0.195 -0.068 0.457 0.42

Aslan,M.,et al (2011) | -0.095 -0.298 0.108 0.44

Atay,A.E.,et al (2014) | 0.621 0.374 0.868 0.43

Atay,A.E.,et al (2013) | 0.621 0.434 0.808 0.45

Aydemir,B.,et al (2016) | 0.203 0.069 0.337 0.46

Bagci, H., et al (2018) | 0.266 -0.085 0.617 0.39

Barat,S.,et al (2018) | 0.789 0.592 0.987 0.45

Barden,A.,et al (2013) | 0.083 -0.152 0.317 0.43

Bartha,J.,et al (2008) | -0.067 -0.621 0.488 0.31

Bartha,J.,et al (2000) | 0.167 -0.274 0.608 0.35

Bawah, A.T,, et aal (2019) | 1.222 0.992 1.453 0.44

Baykus, Y., et al. (2012) | 0.454 -0.168 1.077 0.28

Beigi,A.,et al (2015) | 0.113 -0.287 0.513 0.37

Boghossian,N.,et al (2017) | 0.360 0.175 0.545 0.45

Botta,R.M.,et al (1997) | 0.595 0.297 0.893 0.41

Bugatto，F.,et al (2018) | 0.684 0.181 1.187 0.33

Bullon,P.,et al (2014) | 0.203 -0.112 0.519 0.40

Burlina,S.,et al (2017) | 0.138 -0.204 0.480 0.39

Caglar,G.s.,et al (2011) | 0.190 -0.295 0.675 0.34

Calan, M., et al. (2019) | 0.231 -0.115 0.578 0.39

Camuzcuoglu,H.,et al (2009) | 0.621 0.269 0.973 0.39

Cheng, Y., et al (2010) | 0.534 0.120 0.947 0.37

Cocelli,L.P.,et al (2012) | 0.720 0.390 1.051 0.40

Coskun, A., et al. (2010) | 0.329 -0.027 0.686 0.39

Couch, S.C, et al (1998) | 0.660 0.219 1.101 0.35

Couch,S.C.,et al (1998) | 0.504 0.234 0.773 0.42

Culha,C.,et al (2011) | 0.236 -0.034 0.506 0.42

Davari-Tanha,F.,et al (2008) | 0.474 0.108 0.840 0.38

De La Torre, N.G., et l (2019) | 0.135 0.018 0.253 0.47

Demir , E., et al. (2019) | 0.149 -0.098 0.396 0.43

Demirpence,M.,et al (2016) | -0.467 -1.033 0.098 0.30

Di Cianni,G.,et al (2007) | 0.290 0.243 0.337 0.47

Djelti, F., et al. (2015) | 0.020 -0.035 0.075 0.47

Du,M.K.,et al (2016) | 0.460 0.246 0.674 0.44

Duan, Bide., et al (2020) | 0.530 0.416 0.644 0.47

Dube,E.,et al (2013) | 0.345 -0.432 1.122 0.23

Dudzik,D.,et al (2017) | 0.400 0.069 0.731 0.40

Dudzik,D.,et al (2014) | 0.610 0.226 0.994 0.38

Edu,A.,et al (2016) | 0.104 -0.308 0.516 0.37

Eken,M.K.,et al (2018) | 0.567 0.279 0.855 0.42

El-Beshbishy,H.A.,et al (2015) | 0.847 0.701 0.993 0.46

Erol, O. et al. (2015) | 0.076 -0.251 0.402 0.40

Ersanli, Z.O, et al (1997) | 0.842 0.312 1.372 0.32

Ertuğ, E.Y., et al. (2016) | 0.677 0.276 1.079 0.37

Eslamian, L., et al. (2013) | 0.061 -0.003 0.125 0.47

Ethier-Chiasson, M., et al. (200| 0.910 0.689 1.131 0.44

Fan. Y.C., et al (2020) | 1.650 1.580 1.720 0.47

Franzago, M., et al. (2018) | 0.102 -0.129 0.332 0.44

Gao, Q., et al. (2016) | 0.630 0.177 1.083 0.35

Gao, Y., et al. (2017) | 0.394 0.072 0.716 0.40

Ghafoor, S., et al. (2012) | 0.335 -0.091 0.761 0.36

Giannubilo, S.R., et al. (2011) | 0.040 -0.083 0.163 0.46

Gkiomisi, A., et al. (2013) | 0.710 0.096 1.324 0.29

Grissa, O., et al. (2007) | 0.000 -0.085 0.085 0.47

Grissa,O., et al. (2010) | 0.560 0.487 0.633 0.47

Guimarães, L.O., et al. (2014) | 1.159 1.008 1.311 0.46

Gumus, I.I., et al. (2013) | 0.218 -0.018 0.455 0.43

He, B., et al (2004) | 1.300 0.760 1.840 0.31

He. X.J., et al (2021) | 0.590 0.340 0.840 0.43

Heiskanen, N., et al. (2010) | 0.400 -2.110 2.910 0.04

Hollingsworth, D.R., et al. (198| 1.140 0.299 1.981 0.21

Hornnes, P.J., et al. (1984) | 0.560 -0.283 1.403 0.21

Hossein-nezhad, A., et al. (2010| -0.073 -0.389 0.242 0.40

Hou, W.L., et al. (2016) | 0.200 0.040 0.360 0.46

Houde, A. A., et al. (2013) | 0.170 -0.051 0.391 0.44

Houde, A. A., et al. (2014) | 0.160 -0.052 0.372 0.44

Hsu, H.W., et al. (1997) | 0.390 -0.066 0.845 0.35

Huo, Y., et al (2014) | 0.300 -0.086 0.686 0.38

Huo, Y., et al. (2015) | 0.460 -2.260 3.180 0.03

Idzior-Walus, B., et al. (2008) | 0.800 0.443 1.157 0.39

Iimura, Y., et al. (2015) | 0.274 -0.092 0.640 0.38

Iyidir, O.T., et al. (2015) | 0.508 -0.014 1.030 0.32

Jameshorani, M. et al. (2018) | 0.485 0.249 0.722 0.43

Javadian, P., et al. (2014) | 0.935 -2.833 4.702 0.02

Jia, X.J., et al. (2015) | -0.190 -0.468 0.088 0.42

Kang, J., et al (2019) | 0.208 0.053 0.362 0.46

Kautzky-Willer, A., et al. (1997| 0.132 -1.255 1.520 0.11

Kautzky-Willer, A., et al. (2001| 0.140 -0.207 0.487 0.39

Keskin, F.E., et al. (2015) | -0.175 -0.571 0.221 0.37

Khan, R.. et al. (2013) | 0.233 0.166 0.299 0.47

Khosrowbeygi, A., et al. (2016) | 0.618 -0.038 1.274 0.27

Khosrowbeygi, A., et al. (2018) | 0.387 -0.137 0.912 0.32

Kinalski, M., et al. (2005) | 0.200 -0.140 0.540 0.40

Knopp, R.H., et al. (1980) | 0.203 -0.356 0.762 0.31

Knopp, R.H., et al. (1992) | 0.430 0.282 0.578 0.46

Korkmazer, E., et al. (2015) | 0.666 0.338 0.994 0.40

Kumru, P., et al. (2016) | 0.441 0.199 0.683 0.43

Lacroix, M., et al. (2013) | 0.280 0.049 0.511 0.44

Lehmann, R., et al. (2015) | 0.807 0.608 1.007 0.45

Li, C., et al. (2013) | 0.020 -0.200 0.240 0.44

Li, D.D., et al. (2015) | 0.420 0.186 0.654 0.43

Li, G.H., et al. (2018) | 0.020 -0.203 0.243 0.44

Li, G.H., et al. (2015) | 0.350 0.258 0.442 0.47

Li, H., et al. (2016) | -0.060 -0.125 0.005 0.47

Li, J., et al. (2016) | 1.320 0.865 1.775 0.35

Li, J.Y., et al. (2017) | 0.410 0.102 0.718 0.41

Li, L., et al. (2017) | 0.610 0.391 0.829 0.44

Li, P., et al. (2018) | 0.440 0.271 0.609 0.45

Li, S.M, et al. (2015) | 0.170 -1.517 1.857 0.08

Li, X.M., et al (2015) | 0.527 0.127 0.927 0.37

Li, Y.Y., et al (2015) | 0.570 0.208 0.932 0.39

Liang, Y., et al (2008) | 1.800 1.682 1.918 0.47

Liang, Z.X., et al (2016) | 0.800 0.290 1.310 0.33

Liang, Z.X., et al (2016) | 3.100 2.682 3.518 0.36

Liang, Z.X., et al (2014) | 0.300 0.137 0.463 0.46

Liao, Y., et al. (2018) | 0.950 -1.192 3.092 0.05

Lipu, et al (1997) | 0.000 -0.043 0.043 0.48

Liu, B., et al (2016) | 0.220 0.112 0.328 0.47

Liu, B., et al (2016) | 0.330 -0.277 0.937 0.29

Liu, D., et al (2016) | 0.300 0.127 0.473 0.45

Liu, F., et al (2013) | 1.290 1.046 1.534 0.43

Liu, H., et al (2019) | 0.300 0.078 0.522 0.44

Liu, X., et al (2019) | 0.300 0.106 0.494 0.45

Liu. L., et al (2020) | 0.390 0.021 0.759 0.38

Liu. L., et al (2020) | 0.390 -0.106 0.886 0.33

Liu. L., et al (2020) | 0.230 -0.163 0.623 0.37

Liu. M., et al (2020) | -0.250 -0.357 -0.143 0.47

Liu. P.J. et al (2020) | 0.190 -0.022 0.402 0.44

Liu. T., et al (2020) | 0.510 0.344 0.676 0.45

Liu. Y., et al (2021) | 0.270 -0.036 0.576 0.41

Lou, Y., et al (2014) | 0.268 0.163 0.373 0.47

M, L., et al (2018) | 0.044 -0.013 0.101 0.47

Ma, et al. (2012) | 2.454 2.081 2.826 0.38

Maitland, R. A., et al (2014) | 0.140 -0.462 0.742 0.29

Maple-Brown, L., et al (2012) | 0.300 0.167 0.433 0.46

Marin, A.J., et al (2012) | 0.610 0.400 0.819 0.44

Marseille-Tremblay, C., et al (2| 0.060 -1.271 1.391 0.11

Martino, et al. (2016) | -0.407 -2.009 1.194 0.09

McGrowder, D., et al (2009) | 0.400 -0.038 0.838 0.36

Megia, et al. (2015) | 0.180 -0.033 0.392 0.44

Metzger, B.E., et al (1980) | 1.661 0.745 2.578 0.19

Meyer, B., et al (1996) | 0.350 -0.110 0.810 0.35

Miettinen, H.E., et al (2014) | 0.080 0.002 0.158 0.47

Miettinen, H.E., et al (2018) | 0.180 0.034 0.326 0.46

Mm, W.Q., et al (2014) | 0.290 -0.170 0.750 0.35

Molnar, J., et al (2008) | 0.100 -0.351 0.551 0.35

Montazeri-Najafabady. N., et al | -0.050 -0.159 0.059 0.47

Montelongo, A., et al (1992) | 0.190 -0.087 0.467 0.42

Morimitsu, L.K., et al (2007) | 0.607 0.136 1.079 0.34

Mou Y.Y., et al (2016) | 1.080 0.793 1.367 0.42

Mrizak, I., et al (2013) | 0.410 0.053 0.767 0.39

Mrizak, I., et al (2014) | 0.560 0.487 0.633 0.47

Ning, H., et al (2016) | 0.350 0.265 0.436 0.47

Niu, J.M., et al (2013) | 0.400 0.294 0.506 0.47

Nolan, C.J., et al (1995) | 0.180 -0.039 0.399 0.44

Oiu, C., et al (2007) | 0.508 0.268 0.748 0.43

Onat. T., et al (2021) | 0.260 0.021 0.499 0.43

Ortega-Senovilla, H., et al (20| 0.027 -0.252 0.307 0.42

Ouyang, F.,et al (2002) | 0.320 0.022 0.618 0.41

Pan, B.L., et al (2016) | 1.070 0.832 1.308 0.43

Paradisi, G., et al (2010) | 0.054 -0.328 0.436 0.38

Paradisi, G., et al (2002) | -0.222 -0.316 -0.129 0.47

Pazhohan, A., et al (2019) | 0.386 0.207 0.566 0.45

Pezeshki, B., et al (2019) | 0.085 -0.030 0.200 0.47

Ping, et al. (2012) | 0.350 0.234 0.466 0.47

Prieto-Sanchez, M.T., et al (201| 0.166 -0.224 0.557 0.37

Qiu, Y.H., et al (2016) | -0.165 -0.391 0.062 0.44

Rahman, et al. (2019) | 0.010 -0.322 0.342 0.40

Ranheim, T., et al (2004) | 0.500 -0.207 1.207 0.25

Ren. Z., et al (2020) | 0.020 -0.372 0.412 0.37

Reyes-López, R., et al (2014) | 0.034 -0.196 0.264 0.44

Rizzo, M., et al (2008) | 0.200 -0.220 0.620 0.36

Roca-Rodríguez, et al. (2017) | 3.330 2.128 4.532 0.13

Rojas, I., et al (2002) | 0.220 -0.100 0.540 0.40

Ruchat, et al. (2013) | 0.170 -0.128 0.468 0.41

Ruiz-Palacios, M., et al (2017) | 0.330 -0.129 0.788 0.35

Sanchez-Garcia. A., et al (2020)| 0.000 -0.296 0.296 0.41

Sarkar, P.D., et al (2006) | 0.707 0.541 0.872 0.45

Savona-Ventura, C., et al (2016)| 1.300 1.077 1.523 0.44

Schaefer-Graf, U. M. , et al (20| 0.080 -0.142 0.302 0.44

Scifres, C.M., et al (2011) | 1.140 0.405 1.875 0.24

Shao, J., et al (2015) | 0.180 -0.422 0.782 0.29

Shelley-Jones, D. C. (1993) | 0.638 0.246 1.030 0.37

Shuang, W., et al (2014) | 0.300 0.157 0.443 0.46

Siddiqui, K., et al (2018) | 0.123 -0.474 0.721 0.29

Sobki, S.H., et al (2004) | 0.293 -0.492 1.079 0.23

Soydinc, S., et al (2013) | 0.382 0.014 0.750 0.38

Suntio, K., et al (2010) | 0.400 0.012 0.788 0.38

Sánchez-Vera, I., et al (2007) | 0.340 0.161 0.519 0.45

Takhshid, M.A., et al (2015) | -0.011 -0.430 0.408 0.36

Takhshid, M.A., et al (2015) | -0.006 -0.416 0.405 0.37

Takhshid, M.A., et al (2015) | -0.068 -0.397 0.262 0.40

Tarim, E., et al (2006) | 0.397 0.081 0.713 0.40

Tarim, E., et al (2004) | 0.778 0.586 0.971 0.45

Todoric, J., et al (2013) | 0.247 0.014 0.481 0.43

Trebotic, L.K., et al (2015) | 0.870 0.255 1.484 0.28

Tsai, P.J., et al (2005) | 0.200 -0.161 0.561 0.39

Turek, I.A., et al (2014) | 0.051 -0.187 0.289 0.43

Tuzun, D., et al (2018) | 0.198 -0.069 0.465 0.42

Tönjes, A., et al (2019) | 0.110 -0.214 0.434 0.40

Uebel, K., et al (2014) | 0.300 -0.161 0.761 0.35

Usluoğullari, B., et al. (2017) | -0.173 -0.440 0.095 0.42

Vastagh, I., et al. (2011) | 0.480 0.005 0.955 0.34

Visiedo, F., et al (2013) | 0.104 -0.301 0.509 0.37

Vitoratos, N., et al (2002) | -0.488 -1.158 0.182 0.26

Vural, M., et al. (2012) | 0.321 -0.105 0.746 0.36

Wang, C., et al (2017) | 0.160 0.112 0.208 0.47

Wang, C., et al (2016) | 0.210 0.077 0.343 0.46

Wang, D.Y, et al (2013) | 0.140 -0.106 0.386 0.43

Wang, J., et al (2019) | 0.170 0.100 0.240 0.47

Wang, X., et al (2019) | 0.110 -0.181 0.400 0.41

Wang, Y. Y., et al (2018) | 0.210 0.079 0.341 0.46

Wang, Y.Y., et al (2019) | 0.390 0.151 0.629 0.43

Wani. K., et al (2020) | 0.300 0.146 0.454 0.46

Wei, J.H., et al (2014) | 2.290 2.105 2.475 0.45

Weng. Q., et al (2019) | -0.036 -0.273 0.201 0.43

White, S.L., et al (2016) | 0.200 0.130 0.270 0.47

Whyte, K., et al (2013) | 0.490 0.139 0.841 0.39

Wu, H., et al. (2019) | 0.590 -0.021 1.201 0.29

Wu, K., et al. (2018) | 0.320 0.180 0.460 0.46

Wójcik, M., et al (2015) | 0.436 0.109 0.762 0.40

Wójcik, M., et al (2014) | 0.016 -0.292 0.323 0.41

Xie, R., et al (2000) | 0.900 0.616 1.184 0.42

Xu, M., et al (2015) | 0.430 0.096 0.764 0.40

Xu. H.F., et al (2020) | 0.130 -0.021 0.281 0.46

Yanar, et al. (2019) | 0.300 0.198 0.402 0.47

Yang, X., et al (2017) | 0.390 0.210 0.570 0.45

Yang, Y., et al (2018) | 0.820 0.537 1.103 0.42

Ye, D., et al (2016) | 0.400 0.297 0.503 0.47

Yen, I.W., et al (2019) | 0.234 0.081 0.387 0.46

Yousefzadeh, G., et al (2014) | 0.012 -0.602 0.626 0.28

Yuan, T., et al (2015) | 0.370 -0.058 0.798 0.36

Yuan, T., et al (2014) | 0.500 0.270 0.730 0.44

Yue, C.Y., et al (2018) | 0.740 0.380 1.100 0.39

Zakovicova , et al. (2014) | 0.080 -0.346 0.506 0.36

Zhan, Y., et al (2015) | 0.040 -0.245 0.325 0.42

Zhang, J.W., et al (2017) | 0.850 0.781 0.919 0.47

Zhang, M.Z., et al (2014) | 1.150 0.814 1.486 0.40

Zhang, Y., et al (2017) | 0.820 0.328 1.312 0.33

Zhang, Y., et al (2016) | 1.140 0.670 1.610 0.34

Zhang, Y.S., et al (2018) | 0.200 -0.652 1.052 0.21

Zhang. X.M., et al (2020) | -0.080 -0.186 0.026 0.47

Zhang. Y.Z., et al. (2020) | -0.140 -0.282 0.002 0.46

Zhao, M., et al (2016) | 0.250 0.158 0.342 0.47

Zhao, M., et al (2010) | 0.830 0.655 1.005 0.45

Zheng, D.L., et al (2016) | -0.080 -0.231 0.071 0.46

Zheng, R., et al (2015) | 0.700 0.593 0.807 0.47

Zheng. T., et al (2019) | 0.270 0.204 0.336 0.47

Zhong. L.Q., et al (2020) | 0.250 -0.404 0.904 0.27

Zhou, J., et al. (2018) | 0.410 -0.204 1.024 0.29

Zhou, X., et al (2017) | -0.030 -0.156 0.096 0.46

Zhou, Y., et al (2016) | 0.340 0.190 0.490 0.46

Zhu, J.P., et al (2014) | 0.200 0.074 0.326 0.46

Šimják, et al. (2018) | -0.071 -1.019 0.877 0.18

--------------------------------+----------------------------------------------

Overall, DL | 0.388 0.336 0.439 100.00

-------------------------------------------------------------------------------

Test of overall effect = 0: z = 14.695 p = 0.000
