# Supplementary material for: Association of maternal lipid profile and gestational diabetes mellitus: A systematic review and meta-analysis of 292 studies and 97,880 women
Source: eClinicalMedicine. 2021 Apr 16;34:100830. doi: 10.1016/j.eclinm.2021.100830 (PMC8102708; doi:10.1016/j.eclinm.2021.100830)
Supplement: Supplementary file 4 [file mmc4.docx]

Supplementary Table 3 Summary Weighted Mean Differences of TC from Meta-Analyses

-------------------------------------------------------------------------------

Author (Year) | Effect [95% Conf. Interval] % Weight

--------------------------------+----------------------------------------------

Abo-Elmatty, D. M.,et al (2019) | 1.237 0.913 1.561 0.43

Ademoglu, E., et al (2015) | -0.191 -0.734 0.351 0.36

Akdeniz, F. T.,et al (2017) | 0.484 0.095 0.873 0.41

Akturk, M.,et al (2010) | -0.087 -0.460 0.287 0.42

Akturk, M.,et al (2008) | -0.088 -0.623 0.447 0.36

Al-Ajlan.A.,et al (2018) | 0.200 -0.014 0.414 0.46

Al-Daghri,N.,et al (2018) | 0.300 -0.083 0.683 0.41

Al-Hakeem, M.M., et al (2014) | 0.500 0.299 0.701 0.46

Al-Hakeem, M.M., et al. (2014) | 0.500 -2.562 3.562 0.04

Al-Rubeaan,K.,et al (2014) | -0.170 -0.400 0.060 0.45

Al-Saleh, E., et al. (2007) | 0.336 -0.452 1.125 0.28

Alanbay, I.,et al (2012) | -0.145 -0.567 0.278 0.40

Alattas, O.S., et al (1995) | 2.600 -0.186 5.386 0.05

Alharbi, K.K., et al (2019) | 0.600 0.374 0.826 0.46

Altinova,A.,et al (2007) | 0.336 -0.110 0.783 0.39

Altinova,A.,et al (2015) | 0.163 -0.570 0.896 0.30

Anderwald,C.,et al (2011) | -0.672 -1.789 0.444 0.20

Anghebem-Oliveira,M,I.,et al (20| 0.279 -0.026 0.585 0.44

Anjum,F.,et al (2019) | -0.318 -0.689 0.054 0.42

Aslan,M.,et al (2011) | 0.013 -0.333 0.359 0.42

Atay,A.E.,et al (2014) | 1.629 1.293 1.966 0.43

Atay,A.E.,et al (2013) | 1.629 1.375 1.884 0.45

Aydemir,B.,et al (2016) | 0.521 0.328 0.714 0.46

Bagci, H., et al (2018) | 0.595 0.137 1.052 0.39

Barat,S.,et al (2018) | -0.145 -0.874 0.585 0.30

Bartha,J.,et al (2000) | 0.312 -0.190 0.815 0.37

Bawah, A.T,, et aal (2019) | 2.023 1.562 2.484 0.39

Baykus, Y., et al. (2012) | -0.187 -0.832 0.457 0.33

Beigi,A.,et al (2015) | -0.055 -0.458 0.348 0.41

Boghossian,N.,et al (2017) | 0.090 -0.174 0.354 0.45

Bugatto，F.,et al (2018) | -0.194 -0.856 0.469 0.32

Bullon,P.,et al (2014) | 0.364 -0.252 0.979 0.34

Burlina,S.,et al (2017) | -0.295 -0.966 0.376 0.32

Caglar,G.s.,et al (2011) | 0.197 -0.470 0.863 0.32

Calan, M., et al. (2019) | 0.360 -0.082 0.802 0.39

Camuzcuoglu,H.,et al (2009) | -0.284 -0.688 0.119 0.41

Chen,Y.M.,et al (2017) | -0.456 -0.907 -0.004 0.39

Cheng, Y., et al (2010) | -0.298 -0.664 0.067 0.42

Coskun, A., et al. (2010) | -0.167 -0.603 0.268 0.40

Couch, S.C, et al (1998) | -0.283 -1.290 0.723 0.22

Couch,S.C.,et al (1998) | -0.445 -1.073 0.183 0.33

Culha,C.,et al (2011) | 0.336 -0.057 0.729 0.41

Davari-Tanha,F.,et al (2008) | 0.300 -0.048 0.648 0.42

De La Torre, N.G., et l (2019) | -0.129 -0.335 0.076 0.46

Demir , E., et al. (2019) | 0.178 -0.216 0.573 0.41

Demirpence,M.,et al (2016) | -0.970 -2.161 0.222 0.18

Di Cianni,G.,et al (2007) | -0.010 -0.098 0.078 0.48

Dipla, K., et al. (2017) | 0.100 -0.666 0.866 0.29

Djelti, F., et al. (2015) | 0.040 -0.041 0.121 0.48

Du,M.K.,et al (2016) | 1.120 0.239 2.001 0.26

Duan, Bide., et al (2020) | -0.940 -1.136 -0.744 0.46

Dube,E.,et al (2013) | -1.740 -2.882 -0.598 0.19

Dudzik,D.,et al (2017) | 0.190 -0.347 0.727 0.36

Dudzik,D.,et al (2014) | 0.640 0.060 1.220 0.35

Edu,A.,et al (2016) | -0.013 -0.609 0.584 0.34

Eken,M.K.,et al (2018) | 0.395 -0.038 0.827 0.40

El-Beshbishy,H.A.,et al (2015) | 0.724 0.350 1.098 0.42

Erol, O. et al. (2015) | -0.111 -0.659 0.437 0.36

Ersoy, G.S.. et al. (2017) | 0.339 -1.208 1.885 0.13

Ertuğ, E.Y., et al. (2016) | -0.181 -0.931 0.569 0.29

Eslamian, L., et al. (2013) | 0.085 -0.084 0.255 0.47

Ethier-Chiasson, M., et al. (200| -0.190 -0.429 0.049 0.45

Fan. Y.C., et al (2020) | 1.810 1.600 2.020 0.46

Franzago, M., et al. (2018) | 0.595 0.245 0.944 0.42

Fu,Y., et al (2015) | 0.000 -0.188 0.188 0.46

Gao, Q., et al. (2016) | -0.040 -0.450 0.370 0.40

Gao, Y., et al. (2017) | 0.313 -0.208 0.834 0.37

Ghafoor, S., et al. (2012) | 0.244 -0.462 0.951 0.31

Giannubilo, S.R., et al. (2011) | 0.010 -0.205 0.225 0.46

Gkiomisi, A., et al. (2013) | -0.600 -1.155 -0.045 0.36

Grissa, O., et al. (2007) | -0.960 -1.145 -0.775 0.46

Grissa,O., et al. (2010) | -0.550 -0.687 -0.413 0.47

Guimarães, L.O., et al. (2014) | 0.646 0.395 0.898 0.45

Gumus, I.I., et al. (2013) | 0.741 0.293 1.189 0.39

Guo. Y.Y., et al (2020) | 0.100 -0.118 0.318 0.46

He, B., et al (2004) | 0.500 -0.920 1.920 0.15

He. X.J., et al (2021) | -0.050 -0.312 0.212 0.45

Heiskanen, N., et al. (2010) | -0.500 -3.691 2.691 0.04

Hollingsworth, D.R., et al. (198| -0.259 -1.032 0.515 0.29

Hossein-nezhad, A., et al. (2010| 0.109 -0.204 0.421 0.43

Hou, W., et al. (2018) | 0.000 -0.227 0.227 0.45

Hou, W.L., et al. (2016) | 0.000 -0.145 0.145 0.47

Houde, A. A., et al. (2013) | 0.020 -0.368 0.408 0.41

Houde, A. A., et al. (2014) | -0.120 -0.395 0.155 0.44

Huang, Y., et al. (2016) | -0.830 -1.580 -0.080 0.29

Huang, Y., et al. (2018) | 0.070 -0.129 0.269 0.46

Huo, Y., et al (2014) | -0.400 -0.838 0.038 0.40

Huo, Y., et al. (2015) | -0.260 -2.592 2.072 0.07

Idzior-Walus, B., et al. (2008) | 0.000 -0.560 0.560 0.35

Iimura, Y., et al. (2015) | -0.088 -0.535 0.359 0.39

Iyidir, O.T., et al. (2015) | 0.667 -0.113 1.447 0.28

Jameshorani, M. et al. (2018) | -0.259 -0.683 0.166 0.40

Javadian, P., et al. (2014) | -1.492 -7.230 4.246 0.01

Jia, X.J., et al. (2015) | -0.740 -1.082 -0.398 0.42

Kang, J., et al (2019) | 0.246 -0.036 0.527 0.44

Kautzky-Willer, A., et al. (1997| 1.528 -0.857 3.914 0.06

Kautzky-Willer, A., et al. (2001| -0.790 -1.328 -0.252 0.36

Keskin, F.E., et al. (2015) | -0.437 -0.954 0.080 0.37

Khan, R.. et al. (2013) | 0.283 0.128 0.438 0.47

Khosrowbeygi, A., et al. (2016) | -0.549 -1.412 0.314 0.26

Khosrowbeygi, A., et al. (2018) | -0.435 -1.154 0.284 0.30

Kinalski, M., et al. (2005) | 0.380 -0.122 0.882 0.37

Knopp, R.H., et al. (1980) | -0.052 -0.600 0.496 0.36

Korkmazer, E., et al. (2015) | -0.207 -0.732 0.318 0.37

Koukkou, E., et al. (1996) | -0.480 -1.296 0.336 0.27

Kumru, P., et al. (2016) | 0.574 0.238 0.911 0.43

Lehmann, R., et al. (2015) | -0.238 -0.533 0.057 0.44

Li, C., et al. (2013) | 0.270 -0.029 0.569 0.44

Li, D.D., et al. (2015) | 1.090 0.815 1.365 0.44

Li, G.H., et al. (2015) | 0.230 0.116 0.344 0.47

Li, H., et al. (2016) | 0.230 0.149 0.311 0.48

Li, J., et al. (2016) | 0.600 -0.052 1.252 0.32

Li, J.Y., et al. (2017) | 0.510 0.216 0.804 0.44

Li, L., et al. (2017) | -0.150 -0.389 0.089 0.45

Li, P., et al. (2018) | -0.220 -0.468 0.028 0.45

Li, S.M, et al. (2015) | 0.320 -2.203 2.843 0.06

Li, X.M., et al (2015) | -0.224 -0.650 0.201 0.40

Li, Y.Y., et al (2015) | -0.210 -0.586 0.166 0.41

Liang, Y., et al (2008) | 1.700 1.425 1.975 0.44

Liang, Z.X., et al (2016) | 1.100 0.016 2.184 0.21

Liang, Z.X., et al (2016) | 2.300 1.572 3.028 0.30

Liang, Z.X., et al (2014) | 0.500 0.119 0.881 0.41

Liao, Y., et al. (2018) | 2.170 0.414 3.926 0.11

Lipu, et al (1997) | 0.637 0.452 0.822 0.46

Liu, B., et al (2016) | -0.020 -0.152 0.112 0.47

Liu, D., et al (2016) | 0.280 0.092 0.468 0.46

Liu, F., et al (2013) | 0.770 0.486 1.054 0.44

Liu, H., et al (2019) | 0.500 0.062 0.938 0.39

Liu, X., et al (2019) | 0.200 -0.062 0.462 0.45

Liu. L., et al (2020) | 0.390 -0.603 1.383 0.23

Liu. L., et al (2020) | 0.390 -0.956 1.736 0.16

Liu. L., et al (2020) | 0.120 -0.077 0.317 0.46

Liu. L., et al (2020) | 0.360 -0.642 1.362 0.22

Liu. M., et al (2020) | -0.150 -0.423 0.123 0.44

Liu. P.J. et al (2020) | -0.250 -0.545 0.045 0.44

Liu. T., et al (2020) | -0.060 -0.290 0.170 0.45

Liu. Y., et al (2021) | -0.150 -0.470 0.170 0.43

Lou, Y., et al (2014) | 0.502 0.395 0.609 0.48

M, L., et al (2018) | 2.998 2.868 3.127 0.47

Ma, et al. (2012) | 0.077 -0.400 0.555 0.38

Maitland, R. A., et al (2014) | -0.110 -0.617 0.397 0.37

Makgoba, M., et al (2011) | 0.240 0.020 0.460 0.46

Marin, A.J., et al (2012) | 1.241 0.859 1.624 0.41

Marseille-Tremblay, C., et al (2| 0.250 -1.220 1.720 0.14

McGrowder, D., et al (2009) | 0.700 -0.084 1.484 0.28

Megia, et al. (2015) | -0.136 -0.486 0.214 0.42

Metzger, B.E., et al (1980) | 0.108 -0.946 1.162 0.21

Meyer, B., et al (1996) | -0.070 -0.629 0.489 0.35

Miettinen, H.E., et al (2014) | 0.090 -0.002 0.182 0.48

Miettinen, H.E., et al (2018) | -0.200 -0.350 -0.050 0.47

Mm, W.Q., et al (2014) | -0.210 -0.660 0.240 0.39

Molnar, J., et al (2008) | 0.100 -0.573 0.773 0.32

Montelongo, A., et al (1992) | 0.190 -0.616 0.996 0.28

Morimitsu, L.K., et al (2007) | 0.830 0.081 1.579 0.29

Mou Y.Y., et al (2016) | 0.840 0.588 1.092 0.45

Mrizak, I., et al (2013) | 0.310 -0.451 1.071 0.29

Mrizak, I., et al (2014) | -0.550 -0.687 -0.413 0.47

Naf, S., et al (2012) | -0.090 -0.398 0.218 0.43

Ning, H., et al (2016) | -0.405 -0.538 -0.272 0.47

Niu, J.M., et al (2013) | 0.000 -0.119 0.119 0.47

Oiu, C., et al (2007) | -0.261 -0.559 0.036 0.44

Ouyang, F.,et al (2002) | -0.320 -0.610 -0.030 0.44

Pan, B.L., et al (2016) | 1.430 1.196 1.664 0.45

Paradisi, G., et al (2010) | -0.056 -0.496 0.383 0.39

Paradisi, G., et al (2002) | -0.111 -0.253 0.031 0.47

Pazhohan, A., et al (2019) | 0.181 0.048 0.314 0.47

Pezeshki, B., et al (2019) | 0.133 -0.101 0.368 0.45

Ping, et al. (2012) | -0.090 -0.212 0.032 0.47

Prieto-Sanchez, M.T., et al (201| -0.488 -1.084 0.108 0.34

Qiu, Y.H., et al (2016) | -0.170 -0.382 0.042 0.46

Rahman, et al. (2019) | 0.010 -0.322 0.342 0.43

Ranheim, T., et al (2004) | -0.500 -1.332 0.332 0.27

Ren. Z., et al (2020) | 0.200 -0.345 0.745 0.36

Reyes-López, R., et al (2014) | -0.078 -0.352 0.197 0.44

Rizzo, M., et al (2008) | 0.300 -0.145 0.745 0.39

Roca-Rodríguez, et al. (2017) | -1.010 -1.881 -0.139 0.26

Rojas, I., et al (2002) | 0.190 -0.400 0.780 0.34

Ruchat, et al. (2013) | -0.230 -0.794 0.334 0.35

Sanchez-Garcia. A., et al (2020)| 0.100 -0.199 0.399 0.44

Sarkar, P.D., et al (2006) | 0.499 0.328 0.670 0.47

Savona-Ventura, C., et al (2016)| 0.100 -0.066 0.266 0.47

Savvidou, M., et al (2010) | 0.290 0.100 0.480 0.46

Schaefer-Graf, U. M. , et al (20| 0.110 -0.209 0.429 0.43

Scifres, C.M., et al (2011) | -1.583 -3.234 0.069 0.12

Seghieri, G., et al (2003) | 0.600 -0.055 1.255 0.32

Shao, J., et al (2015) | 0.050 -0.708 0.808 0.29

Shelley-Jones, D. C. (1993) | -0.775 -1.742 0.191 0.23

Shuang, W., et al (2014) | 0.200 0.020 0.380 0.46

Siddiqui, K., et al (2018) | -0.207 -0.996 0.582 0.28

Simon-Muela, I., et al (2015) | -0.250 -0.610 0.110 0.42

Sobki, S.H., et al (2004) | -0.073 -0.702 0.556 0.33

Su, Y.X., et al (2010) | -0.100 -0.510 0.310 0.40

Suntio, K., et al (2010) | -0.500 -1.012 0.012 0.37

Sánchez-Vera, I., et al (2007) | 0.900 0.334 1.466 0.35

Takhshid, M.A., et al (2015) | -0.181 -0.614 0.252 0.40

Takhshid, M.A., et al (2015) | -0.240 -0.670 0.189 0.40

Takhshid, M.A., et al (2015) | -0.491 -0.941 -0.042 0.39

Tarim, E., et al (2006) | 0.325 -0.129 0.780 0.39

Tarim, E., et al (2004) | 0.487 0.108 0.866 0.41

Todoric, J., et al (2013) | -0.378 -0.722 -0.033 0.42

Todoric, J., et al (2013) | -0.370 -0.958 0.218 0.35

Trebotic, L.K., et al (2015) | 1.029 0.156 1.902 0.26

Tsai, P.J., et al (2005) | -0.400 -0.742 -0.058 0.42

Turek, I.A., et al (2014) | -0.167 -0.524 0.191 0.42

Tönjes, A., et al (2019) | 0.240 -0.198 0.678 0.39

Uebel, K., et al (2014) | -0.328 -1.065 0.408 0.30

Vastagh, I., et al. (2011) | 1.050 0.554 1.546 0.38

Visiedo, F., et al (2013) | -0.512 -1.730 0.706 0.18

Vural, M., et al. (2012) | 0.204 -0.242 0.650 0.39

Wang, C., et al (2017) | 0.150 0.093 0.207 0.48

Wang, C., et al (2016) | 0.130 0.075 0.185 0.48

Wang, D.Y, et al (2013) | 0.320 -0.102 0.742 0.40

Wang, H.Y., et al (2019) | 0.315 0.015 0.615 0.44

Wang, J., et al (2019) | 0.030 -0.055 0.115 0.48

Wang, X., et al (2019) | 0.080 -0.230 0.390 0.43

Wang, Y. Y., et al (2018) | 0.220 0.034 0.406 0.46

Wang, Y.Y., et al (2019) | 0.110 -0.181 0.401 0.44

Wani. K., et al (2020) | 0.400 0.129 0.671 0.44

Wei, J.H., et al (2014) | 2.230 1.999 2.461 0.45

Weng. Q., et al (2019) | -0.246 -0.481 -0.011 0.45

White, S.L., et al (2016) | 0.000 -0.140 0.140 0.47

Whyte, K., et al (2013) | 0.230 -0.197 0.657 0.40

Wu, H., et al. (2019) | 0.020 -0.359 0.399 0.41

Wu, K., et al. (2018) | 0.110 -0.033 0.253 0.47

Wójcik, M., et al (2015) | 0.305 -0.168 0.778 0.38

Wójcik, M., et al (2014) | -0.207 -0.575 0.162 0.42

Xie, R., et al (2000) | -0.300 -0.655 0.055 0.42

Xu, M., et al (2015) | -0.030 -0.431 0.371 0.41

Xu. H.F., et al (2020) | 1.090 0.834 1.346 0.45

Yanar, et al. (2019) | 0.300 0.118 0.482 0.46

Yang, X., et al (2017) | -0.740 -0.903 -0.577 0.47

Yang, Y., et al (2018) | 0.530 0.322 0.738 0.46

Ye, D., et al (2016) | 0.100 -0.021 0.221 0.47

Yen, I.W., et al (2019) | 0.209 -0.092 0.511 0.44

Yousefzadeh, G., et al (2014) | 0.220 -0.451 0.890 0.32

Yuan, T., et al (2015) | -0.090 -0.702 0.522 0.34

Yuan, T., et al (2014) | -0.100 -0.346 0.146 0.45

Yue, C.Y., et al (2018) | 0.030 -0.182 0.242 0.46

Zakovicova , et al. (2014) | -0.100 -0.535 0.335 0.40

Zhan, Y., et al (2015) | 0.260 -0.132 0.652 0.41

Zhang, J.W., et al (2017) | 0.170 -0.029 0.369 0.46

Zhang, M.Z., et al (2014) | 1.180 0.776 1.584 0.41

Zhang, Y., et al (2017) | 0.020 -0.350 0.390 0.42

Zhang, Y., et al (2016) | -0.040 -0.396 0.316 0.42

Zhang, Y.S., et al (2018) | 0.860 0.412 1.308 0.39

Zhang. X.M., et al (2020) | 0.160 0.014 0.306 0.47

Zhang. Y.Z., et al. (2020) | -0.010 -0.142 0.122 0.47

Zhao, M., et al (2016) | 0.120 0.018 0.222 0.48

Zhao, M., et al (2010) | -0.520 -0.737 -0.303 0.46

Zheng, D.L., et al (2016) | -0.160 -0.430 0.110 0.44

Zheng, R., et al (2015) | 0.170 -0.010 0.350 0.46

Zheng. T., et al (2019) | 0.080 0.011 0.149 0.48

Zhong. L.Q., et al (2020) | 0.240 -0.290 0.770 0.36

Zhou, J., et al. (2018) | 0.070 -0.398 0.538 0.39

Zhou, X., et al (2017) | 0.030 -0.205 0.265 0.45

Zhou, Y., et al (2016) | 0.180 0.127 0.233 0.48

Zhou. M., et al (2020) | -0.060 -0.244 0.124 0.46

Zhu, J.P., et al (2014) | 0.110 -0.054 0.274 0.47

de Melo, S.F., et al. (2015) | 1.020 0.766 1.274 0.45

dos Santos-Weiss,I.C.,et al (201| 0.200 -0.111 0.511 0.43

Šimják, et al. (2018) | -0.590 -1.782 0.602 0.18

--------------------------------+----------------------------------------------

Overall, DL | 0.149 0.084 0.214 100.00

-------------------------------------------------------------------------------

Test of overall effect = 0: z = 4.494 p = 0.000
