# Supplementary material for: Association of maternal lipid profile and gestational diabetes mellitus: A systematic review and meta-analysis of 292 studies and 97,880 women
Source: eClinicalMedicine. 2021 Apr 16;34:100830. doi: 10.1016/j.eclinm.2021.100830 (PMC8102708; doi:10.1016/j.eclinm.2021.100830)
Supplement: Supplementary file 5 [file mmc5.docx]

Supplementary Table 4 Summary Weighted Mean Differences of HDL-C from Meta-Analyses

-------------------------------------------------------------------------------

Author (Year) | Effect [95% Conf. Interval] % Weight

--------------------------------+----------------------------------------------

Abo-Elmatty, D. M.,et al (2019) | -0.245 -0.326 -0.164 0.53

Ademoglu, E., et al (2015) | -0.310 -0.472 -0.149 0.43

Akdeniz, F. T.,et al (2017) | -0.294 -0.430 -0.157 0.47

Akturk, M.,et al (2010) | -0.119 -0.283 0.044 0.43

Akturk, M.,et al (2008) | -0.026 -0.232 0.180 0.37

Al-Ajlan.A.,et al (2018) | 0.000 -0.064 0.064 0.55

Al-Daghri,N.,et al (2018) | 0.100 -0.027 0.227 0.48

Al-Hakeem, M.M., et al (2014) | 0.280 0.221 0.339 0.56

Al-Hakeem, M.M., et al. (2014) | 0.280 -0.601 1.161 0.05

Al-Rubeaan,K.,et al (2014) | -0.080 -0.136 -0.024 0.56

Alanbay, I.,et al (2012) | -0.243 -0.362 -0.125 0.49

Alharbi, K.K., et al (2019) | 0.300 0.238 0.362 0.55

Altinova,A.,et al (2007) | -0.106 -0.325 0.113 0.35

Altinova,A.,et al (2015) | -0.295 -0.464 -0.126 0.42

Anderwald,C.,et al (2011) | -0.362 -0.955 0.231 0.10

Anghebem-Oliveira,M,I.,et al (20| 0.036 -0.055 0.127 0.52

Anjum,F.,et al (2019) | -0.339 -0.479 -0.199 0.46

Aslan,M.,et al (2011) | -0.111 -0.282 0.059 0.42

Atay,A.E.,et al (2014) | -0.155 -0.214 -0.097 0.56

Atay,A.E.,et al (2013) | -0.155 -0.199 -0.111 0.57

Aydemir,B.,et al (2016) | 0.027 -0.011 0.066 0.57

Bagci, H., et al (2018) | 0.079 -0.059 0.218 0.46

Barat,S.,et al (2018) | -0.336 -0.484 -0.188 0.45

Barden,A.,et al (2013) | 2.080 1.927 2.233 0.44

Bartha, J.L., et al (2000) | 0.481 -0.075 1.037 0.11

Bartha,J.,et al (2008) | 0.290 -0.009 0.589 0.27

Bartha,J.,et al (2000) | 0.492 0.217 0.766 0.29

Bawah, A.T,, et aal (2019) | -0.158 -0.467 0.152 0.26

Baykus, Y., et al. (2012) | -0.106 -0.329 0.117 0.35

Beigi,A.,et al (2015) | -0.062 -0.201 0.078 0.46

Bo,S.,et al (2009) | -0.200 -0.250 -0.150 0.56

Boghossian,N.,et al (2017) | -0.100 -0.185 -0.015 0.53

Bugatto，F.,et al (2018) | -0.140 -0.446 0.167 0.26

Bullon,P.,et al (2014) | 0.045 -0.154 0.245 0.38

Burlina,S.,et al (2017) | -0.297 -0.547 -0.048 0.32

Caglar,G.s.,et al (2011) | -0.202 -0.511 0.108 0.26

Calan, M., et al. (2019) | -0.068 -0.200 0.065 0.47

Camuzcuoglu,H.,et al (2009) | -0.398 -0.521 -0.276 0.49

Chen,Y.M.,et al (2017) | -0.139 -0.349 0.071 0.37

Cheng, Y., et al (2010) | -0.169 -0.316 -0.022 0.45

Cocelli,L.P.,et al (2012) | -0.375 -0.460 -0.290 0.53

Coskun, A., et al. (2010) | -0.021 -0.180 0.138 0.44

Couch, S.C, et al (1998) | 0.020 -0.289 0.329 0.26

Culha,C.,et al (2011) | -0.163 -0.347 0.021 0.40

Davari-Tanha,F.,et al (2008) | -0.272 -0.434 -0.109 0.43

Demir , E., et al. (2019) | -0.054 -0.178 0.070 0.48

Demirpence,M.,et al (2016) | -0.018 -0.351 0.315 0.24

Di Cianni,G.,et al (2007) | -0.080 -0.106 -0.054 0.58

Dipla, K., et al. (2017) | -0.200 -0.420 0.020 0.35

Du,M.K.,et al (2016) | -0.520 -0.707 -0.333 0.40

Dube,E.,et al (2013) | -0.370 -0.701 -0.039 0.24

Dudzik,D.,et al (2017) | -0.160 -0.401 0.081 0.33

Dudzik,D.,et al (2014) | 0.100 -0.170 0.370 0.30

Edu,A.,et al (2016) | 0.330 0.175 0.485 0.44

Eken,M.K.,et al (2018) | -0.024 -0.113 0.066 0.53

El-Beshbishy,H.A.,et al (2015) | -0.103 -0.215 0.008 0.50

Erol, O. et al. (2015) | -0.047 -0.190 0.097 0.46

Ertuğ, E.Y., et al. (2016) | -0.129 -0.348 0.089 0.36

Eslamian, L., et al. (2013) | -0.000 -0.022 0.022 0.58

Ethier-Chiasson, M., et al. (200| -0.230 -0.321 -0.139 0.52

Fan. Y.C., et al (2020) | -0.630 -0.700 -0.560 0.55

Franzago, M., et al. (2018) | 0.000 -0.125 0.125 0.48

Fu,Y., et al (2015) | -0.040 -0.115 0.035 0.54

Gao, Q., et al. (2016) | -0.130 -0.256 -0.004 0.48

Gao, Y., et al. (2017) | -0.202 -0.471 0.068 0.30

Ghafoor, S., et al. (2012) | -0.264 -0.382 -0.145 0.49

Gkiomisi, A., et al. (2013) | -0.180 -0.361 0.001 0.41

Grissa,O., et al. (2010) | -0.050 -0.122 0.022 0.54

Guimarães, L.O., et al. (2014) | 0.096 0.017 0.174 0.54

Gumus, I.I., et al. (2013) | -0.112 -0.252 0.028 0.46

He, B., et al (2004) | 0.000 -0.211 0.211 0.37

He. X.J., et al (2021) | -0.040 -0.118 0.038 0.54

Heiskanen, N., et al. (2010) | -0.200 -1.586 1.186 0.02

Hossein-nezhad, A., et al. (2010| -0.018 -0.119 0.083 0.51

Hou, W., et al. (2018) | -0.100 -0.196 -0.004 0.52

Hou, W.L., et al. (2016) | 0.000 -0.060 0.060 0.55

Houde, A. A., et al. (2013) | 0.060 -0.085 0.205 0.45

Houde, A. A., et al. (2014) | 0.040 -0.093 0.173 0.47

Huang, Y., et al. (2016) | -0.380 -0.643 -0.117 0.30

Huo, Y., et al (2014) | -0.100 -0.306 0.106 0.37

Huo, Y., et al. (2015) | -0.080 -0.760 0.600 0.08

Idzior-Walus, B., et al. (2008) | -0.300 -0.541 -0.059 0.33

Iimura, Y., et al. (2015) | -0.034 -0.167 0.099 0.47

Iyidir, O.T., et al. (2015) | -0.028 -0.238 0.181 0.37

Jaffe. A., et al (2020) | -0.069 -0.127 -0.010 0.56

Jameshorani, M. et al. (2018) | -0.318 -0.460 -0.176 0.46

Javadian, P., et al. (2014) | -0.917 -2.991 1.157 0.01

Kang, J., et al (2019) | -0.026 -0.151 0.100 0.48

Kautzky-Willer, A., et al. (1997| -0.153 -0.651 0.346 0.14

Kautzky-Willer, A., et al. (2001| -0.240 -0.436 -0.044 0.39

Keskin, F.E., et al. (2015) | -0.137 -0.295 0.021 0.44

Khan, R.. et al. (2013) | -0.028 -0.090 0.034 0.55

Khosrowbeygi, A., et al. (2016) | -0.651 -0.849 -0.453 0.38

Khosrowbeygi, A., et al. (2018) | -0.453 -0.656 -0.250 0.38

Kinalski, M., et al. (2005) | -0.020 -0.144 0.104 0.48

Korkmazer, E., et al. (2015) | -0.129 -0.295 0.036 0.43

Koukkou, E., et al. (1996) | -0.010 -0.226 0.206 0.36

Kumru, P., et al. (2016) | -0.127 -0.244 -0.010 0.49

Lehmann, R., et al. (2015) | -0.352 -0.440 -0.264 0.53

Li, C., et al. (2013) | -0.090 -0.204 0.024 0.50

Li, D.D., et al. (2015) | -0.390 -0.536 -0.244 0.45

Li, G.H., et al. (2018) | -0.060 -0.162 0.042 0.51

Li, G.H., et al. (2015) | -0.130 -0.181 -0.079 0.56

Li, H., et al. (2016) | 0.070 0.030 0.110 0.57

Li, J., et al. (2016) | 0.100 -0.078 0.278 0.41

Li, J.Y., et al. (2017) | 0.090 -0.033 0.213 0.48

Li, P., et al. (2018) | -0.250 -0.333 -0.167 0.53

Li, S.M, et al. (2015) | 1.600 0.071 3.129 0.02

Li, X.M., et al (2015) | -0.040 -0.699 0.620 0.09

Li, Y.Y., et al (2015) | -0.350 -0.560 -0.140 0.37

Li. F., et al (2020) | 0.130 -0.144 0.404 0.29

Liang, Z.X., et al (2016) | -0.500 -0.794 -0.206 0.27

Liao, Y., et al. (2018) | 0.970 0.399 1.541 0.11

Lipu, et al (1997) | -0.312 -0.450 -0.174 0.46

Liu, B., et al (2016) | -0.030 -0.075 0.015 0.57

Liu, B., et al (2016) | 0.000 -0.199 0.199 0.38

Liu, D., et al (2016) | 0.000 -0.095 0.095 0.52

Liu, H., et al (2019) | 0.100 -0.085 0.285 0.40

Liu. L., et al (2020) | -0.090 -0.212 0.032 0.49

Liu. L., et al (2020) | -0.090 -0.251 0.071 0.43

Liu. L., et al (2020) | -0.070 -0.144 0.004 0.54

Liu. L., et al (2020) | -0.080 -0.211 0.051 0.47

Liu. M., et al (2020) | -0.100 -0.287 0.087 0.40

Liu. P.J. et al (2020) | -0.030 -0.115 0.055 0.53

Liu. T., et al (2020) | -0.420 -0.610 -0.230 0.39

Liu. Y., et al (2021) | -0.310 -0.431 -0.189 0.49

Lou, Y., et al (2014) | 0.010 -0.021 0.041 0.57

M, L., et al (2018) | 0.083 0.041 0.125 0.57

Ma, et al. (2012) | 0.006 -0.104 0.117 0.50

Maitland, R. A., et al (2014) | -0.070 -0.627 0.487 0.11

Maple-Brown, L., et al (2012) | -0.100 -0.166 -0.034 0.55

Marseille-Tremblay, C., et al (2| -0.180 -0.540 0.180 0.21

McGrowder, D., et al (2009) | -0.200 -0.422 0.022 0.35

Megia, et al. (2015) | -0.043 -0.149 0.063 0.51

Miettinen, H.E., et al (2014) | -0.030 -0.070 0.010 0.57

Miettinen, H.E., et al (2018) | -0.100 -0.189 -0.011 0.53

Mm, W.Q., et al (2014) | -0.138 -0.278 0.003 0.46

Molnar, J., et al (2008) | -0.200 -0.461 0.061 0.31

Montelongo, A., et al (1992) | -0.090 -0.574 0.394 0.14

Morimitsu, L.K., et al (2007) | 0.028 -0.252 0.309 0.28

Mou Y.Y., et al (2016) | -0.250 -0.347 -0.153 0.52

Mrizak, I., et al (2013) | -0.160 -0.407 0.087 0.32

Mrizak, I., et al (2014) | -0.050 -0.122 0.022 0.54

Naf, S., et al (2012) | -0.060 -0.153 0.033 0.52

Niu, J.M., et al (2013) | -0.100 -0.138 -0.062 0.57

Oiu, C., et al (2007) | -0.178 -0.275 -0.082 0.52

Pan, B.L., et al (2016) | -0.100 -0.222 0.022 0.49

Paradisi, G., et al (2010) | 0.082 -0.195 0.360 0.29

Paradisi, G., et al (2002) | 0.160 0.113 0.207 0.56

Pezeshki, B., et al (2019) | -0.025 -0.097 0.048 0.54

Ping, et al. (2012) | -0.060 -0.113 -0.007 0.56

Prieto-Sanchez, M.T., et al (201| -0.194 -0.412 0.023 0.36

Qiu, Y.H., et al (2016) | -0.074 -0.129 -0.018 0.56

Ranheim, T., et al (2004) | -0.200 -0.436 0.036 0.33

Ren. Z., et al (2020) | -0.250 -0.800 0.300 0.12

Reyes-López, R., et al (2014) | -0.078 -0.148 -0.007 0.55

Rizzo, M., et al (2008) | -0.100 -0.350 0.150 0.32

Roca-Rodríguez, et al. (2017) | -0.640 -0.958 -0.322 0.25

Rojas, I., et al (2002) | -0.130 -0.353 0.093 0.35

Ruchat, et al. (2013) | -0.030 -0.189 0.129 0.44

Sarkar, P.D., et al (2006) | -0.150 -0.203 -0.097 0.56

Savona-Ventura, C., et al (2016)| 0.100 0.035 0.165 0.55

Savvidou, M., et al (2010) | -0.130 -0.211 -0.049 0.54

Seghieri, G., et al (2003) | 0.000 -0.213 0.213 0.36

Shuang, W., et al (2014) | 0.000 -0.096 0.096 0.52

Siddiqui, K., et al (2018) | 0.160 -0.084 0.405 0.32

Simon-Muela, I., et al (2015) | -0.030 -0.174 0.114 0.46

Sobki, S.H., et al (2004) | -0.062 -0.235 0.111 0.42

Soydinc, S., et al (2013) | 0.166 0.025 0.308 0.46

Sreckovic, I., et al (2014) | -0.621 -1.097 -0.145 0.15

Su, Y.X., et al (2010) | 0.000 -0.125 0.125 0.48

Suntio, K., et al (2010) | -0.200 -0.420 0.020 0.35

Takhshid, M.A., et al (2015) | 0.026 -0.086 0.138 0.50

Takhshid, M.A., et al (2015) | -0.005 -0.115 0.104 0.50

Takhshid, M.A., et al (2015) | -0.052 -0.152 0.049 0.51

Tarim, E., et al (2006) | 0.048 -0.207 0.303 0.31

Tarim, E., et al (2004) | -0.074 -0.220 0.072 0.45

Todoric, J., et al (2013) | -0.176 -0.298 -0.053 0.48

Trebotic, L.K., et al (2015) | -0.121 -0.385 0.144 0.30

Tsai, P.J., et al (2005) | 0.000 -0.114 0.114 0.50

Turek, I.A., et al (2014) | -0.188 -0.327 -0.049 0.46

Tuzun, D., et al (2018) | -0.009 -0.184 0.166 0.41

Tönjes, A., et al (2019) | -0.050 -0.188 0.088 0.46

Uebel, K., et al (2014) | 0.048 -0.184 0.280 0.34

Vastagh, I., et al. (2011) | -0.140 -0.293 0.013 0.44

Visiedo, F., et al (2013) | -1.005 -1.764 -0.246 0.07

Vural, M., et al. (2012) | -0.119 -0.260 0.022 0.46

Wang, C., et al (2017) | -0.050 -0.085 -0.015 0.57

Wang, D.Y, et al (2013) | 0.090 -0.051 0.231 0.46

Wang, J., et al (2019) | -0.060 -0.093 -0.027 0.57

Wang, X., et al (2019) | -0.140 -0.253 -0.027 0.50

Wang, Y. Y., et al (2018) | -0.020 -0.091 0.051 0.55

Wang, Y.Y., et al (2019) | -0.130 -0.256 -0.005 0.48

Wani. K., et al (2020) | -0.100 -0.177 -0.023 0.54

Weng. Q., et al (2019) | -0.062 -0.150 0.026 0.53

Whyte, K., et al (2013) | -0.150 -0.309 0.009 0.44

Wu, H., et al. (2019) | -0.080 -0.200 0.040 0.49

Wu, K., et al. (2018) | 0.030 -0.005 0.065 0.57

Wójcik, M., et al (2015) | -0.209 -0.385 -0.034 0.41

Wójcik, M., et al (2014) | -0.191 -0.342 -0.041 0.45

Xu, M., et al (2015) | -0.020 -0.157 0.117 0.47

Yanar, et al. (2019) | 0.000 -0.058 0.058 0.56

Yang, X., et al (2017) | -0.280 -0.338 -0.222 0.56

Yang, Y., et al (2018) | -0.160 -0.232 -0.088 0.54

Ye, D., et al (2016) | -0.100 -0.172 -0.028 0.54

Yen, I.W., et al (2019) | -0.067 -0.187 0.053 0.49

Yousefzadeh, G., et al (2014) | 0.132 -0.002 0.266 0.47

Yuan, T., et al (2015) | 0.030 -0.208 0.268 0.33

Yuan, T., et al (2014) | 0.000 -0.095 0.095 0.52

Yue, C.Y., et al (2018) | 0.000 -0.044 0.044 0.57

Zakovicova , et al. (2014) | 0.060 -0.109 0.229 0.42

Zhan, Y., et al (2015) | -0.120 -0.258 0.018 0.46

Zhang, J.W., et al (2017) | -0.270 -0.329 -0.211 0.56

Zhang, Y., et al (2017) | -0.020 -0.341 0.301 0.25

Zhang, Y., et al (2016) | -0.080 -0.261 0.101 0.40

Zhang, Y.S., et al (2018) | 0.080 -0.048 0.208 0.48

Zhang. X.M., et al (2020) | -0.010 -0.059 0.039 0.56

Zhang. Y.Z., et al. (2020) | -0.040 -0.089 0.009 0.56

Zhao, M., et al (2016) | -0.090 -0.155 -0.025 0.55

Zhao, M., et al (2010) | -0.270 -0.334 -0.206 0.55

Zheng, D.L., et al (2016) | -0.180 -0.375 0.015 0.39

Zheng, R., et al (2015) | -0.050 -0.136 0.036 0.53

Zheng. T., et al (2019) | -0.170 -0.216 -0.124 0.57

Zhong. L.Q., et al (2020) | 0.030 -0.144 0.204 0.41

Zhou, J., et al. (2018) | 0.040 -0.341 0.421 0.20

Zhou, X., et al (2017) | -0.040 -0.120 0.040 0.54

Zhou, Y., et al (2016) | -0.040 -0.062 -0.018 0.58

Zhou. M., et al (2020) | -0.020 -0.097 0.057 0.54

Zhu, J.P., et al (2014) | 0.010 -0.086 0.106 0.52

de Melo, S.F., et al. (2015) | 0.240 0.179 0.301 0.55

dos Santos-Weiss,I.C.,et al (201| -0.200 -0.315 -0.085 0.49

Šimják, et al. (2018) | -0.090 -0.534 0.354 0.16

--------------------------------+----------------------------------------------

Overall, DL | -0.079 -0.100 -0.058 100.00

-------------------------------------------------------------------------------

Test of overall effect = 0: z = -7.396 p = 0.000
