# Supplementary material for: Association of maternal lipid profile and gestational diabetes mellitus: A systematic review and meta-analysis of 292 studies and 97,880 women
Source: eClinicalMedicine. 2021 Apr 16;34:100830. doi: 10.1016/j.eclinm.2021.100830 (PMC8102708; doi:10.1016/j.eclinm.2021.100830)
Supplement: Supplementary file 6 [file mmc6.docx]

Supplementary Table 5 Summary Weighted Mean Differences of LDL-C from Meta-Analyses

-------------------------------------------------------------------------------

Author (Year) | Effect [95% Conf. Interval] % Weight

--------------------------------+----------------------------------------------

Abo-Elmatty, D. M.,et al (2019) | 1.426 1.096 1.757 0.47

Ademoglu, E., et al (2015) | 0.059 -0.424 0.543 0.40

Akdeniz, F. T.,et al (2017) | 0.299 -0.013 0.610 0.47

Akturk, M.,et al (2010) | -0.230 -0.626 0.166 0.44

Akturk, M.,et al (2008) | -0.243 -0.783 0.297 0.38

Al-Ajlan.A.,et al (2018) | 0.100 -0.087 0.287 0.51

Al-Daghri,N.,et al (2018) | 0.100 -0.191 0.391 0.48

Al-Hakeem, M.M., et al (2014) | 0.000 -0.172 0.172 0.52

Al-Hakeem, M.M., et al. (2014) | 0.000 -2.677 2.677 0.05

Al-Rubeaan,K.,et al (2014) | -0.050 -0.247 0.147 0.51

Alanbay, I.,et al (2012) | -0.238 -0.621 0.145 0.45

Alharbi, K.K., et al (2019) | 0.000 -0.186 0.186 0.51

Altinova,A.,et al (2007) | 0.186 -0.176 0.549 0.45

Altinova,A.,et al (2015) | -0.047 -0.612 0.519 0.37

Anghebem-Oliveira,M,I.,et al (20| -0.191 -0.448 0.066 0.49

Anjum,F.,et al (2019) | -0.064 -0.374 0.245 0.47

Aslan,M.,et al (2011) | 0.031 -0.318 0.380 0.46

Atay,A.E.,et al (2014) | 1.655 1.349 1.961 0.48

Atay,A.E.,et al (2013) | 1.655 1.424 1.886 0.50

Aydemir,B.,et al (2016) | 0.021 -0.055 0.098 0.54

Bagci, H., et al (2018) | -0.035 -0.368 0.297 0.47

Barat,S.,et al (2018) | 0.006 -0.250 0.263 0.49

Bartha, J.L., et al (2000) | -0.724 -1.379 -0.069 0.33

Bartha,J.,et al (2000) | -0.244 -0.771 0.283 0.39

Bawah, A.T,, et aal (2019) | 1.424 0.925 1.923 0.40

Baykus, Y., et al. (2012) | 0.145 -0.428 0.717 0.37

Beigi,A.,et al (2015) | -0.039 -0.296 0.219 0.49

Boghossian,N.,et al (2017) | 0.000 -0.234 0.234 0.50

Bugatto，F.,et al (2018) | -0.078 -0.636 0.481 0.37

Bullon,P.,et al (2014) | 0.204 -0.279 0.686 0.40

Burlina,S.,et al (2017) | -0.034 -0.615 0.548 0.36

Caglar,G.s.,et al (2011) | 0.085 -0.550 0.721 0.34

Calan, M., et al. (2019) | 0.322 -0.084 0.728 0.44

Camuzcuoglu,H.,et al (2009) | 0.776 0.471 1.080 0.48

Chen,Y.M.,et al (2017) | -0.476 -0.842 -0.109 0.45

Cheng, Y., et al (2010) | -0.364 -0.609 -0.119 0.50

Cocelli,L.P.,et al (2012) | 0.802 0.000 1.603 0.28

Correa,P.J.,et al (2019) | 0.636 0.177 1.095 0.41

Coskun, A., et al. (2010) | 0.372 -0.101 0.845 0.41

Couch, S.C, et al (1998) | -0.124 -0.888 0.641 0.29

Davari-Tanha,F.,et al (2008) | 0.155 -0.125 0.436 0.49

Demir , E., et al. (2019) | 0.103 -0.233 0.440 0.46

Demirpence,M.,et al (2016) | -0.740 -1.772 0.292 0.21

Di Cianni,G.,et al (2007) | -0.030 -0.116 0.056 0.53

Dipla, K., et al. (2017) | 0.000 -0.728 0.728 0.31

Du,M.K.,et al (2016) | 0.410 0.070 0.750 0.46

Dube,E.,et al (2013) | -1.605 -2.609 -0.601 0.22

Dudzik,D.,et al (2017) | 0.290 -0.259 0.839 0.38

Dudzik,D.,et al (2014) | 0.240 -0.280 0.760 0.39

Edu,A.,et al (2016) | 0.472 -0.106 1.050 0.36

Eken,M.K.,et al (2018) | 0.202 -0.134 0.539 0.46

El-Beshbishy,H.A.,et al (2015) | 1.164 0.822 1.506 0.46

Erol, O. et al. (2015) | 0.003 -0.473 0.478 0.41

Ertuğ, E.Y., et al. (2016) | -0.440 -1.144 0.265 0.32

Eslamian, L., et al. (2013) | 0.096 -0.054 0.247 0.52

Ethier-Chiasson, M., et al. (200| -0.370 -0.532 -0.208 0.52

Fan. Y.C., et al (2020) | 1.330 1.287 1.373 0.54

Franzago, M., et al. (2018) | 1.293 0.853 1.733 0.42

Gao, Q., et al. (2016) | -0.240 -0.526 0.046 0.48

Gao, Y., et al. (2017) | -0.308 -0.584 -0.031 0.49

Ghafoor, S., et al. (2012) | 0.343 -0.303 0.990 0.34

Gkiomisi, A., et al. (2013) | -0.600 -1.099 -0.101 0.40

Grissa,O., et al. (2010) | -0.450 -0.582 -0.318 0.53

Guimarães, L.O., et al. (2014) | 0.049 -0.169 0.267 0.51

Gumus, I.I., et al. (2013) | 0.571 0.225 0.917 0.46

Guo. Y.Y., et al (2020) | 0.300 0.097 0.503 0.51

He, B., et al (2004) | 0.000 -0.539 0.539 0.38

He. X.J., et al (2021) | -0.090 -0.236 0.056 0.52

Heiskanen, N., et al. (2010) | -0.600 -3.514 2.314 0.04

Hossein-nezhad, A., et al. (2010| -0.059 -0.274 0.155 0.51

Hou, W.L., et al. (2016) | 0.000 -0.105 0.105 0.53

Houde, A. A., et al. (2013) | 0.090 -0.234 0.414 0.47

Houde, A. A., et al. (2014) | -0.150 -0.389 0.089 0.50

Huang, Y., et al. (2016) | -0.490 -1.077 0.097 0.36

Huo, Y., et al (2014) | -0.100 -0.462 0.262 0.45

Huo, Y., et al. (2015) | -0.160 -2.157 1.837 0.08

Idzior-Walus, B., et al. (2008) | -0.200 -0.682 0.282 0.41

Iimura, Y., et al. (2015) | 0.031 -0.350 0.412 0.45

Iyidir, O.T., et al. (2015) | 0.424 -0.236 1.085 0.33

Jameshorani, M. et al. (2018) | -0.543 -0.851 -0.235 0.48

Javadian, P., et al. (2014) | 1.298 -0.081 2.677 0.14

Kang, J., et al (2019) | 6.808 -0.123 13.739 0.01

Kautzky-Willer, A., et al. (1997| 0.714 -0.010 1.438 0.31

Kautzky-Willer, A., et al. (2001| -0.560 -1.028 -0.092 0.41

Keskin, F.E., et al. (2015) | -0.163 -0.628 0.302 0.41

Khan, R.. et al. (2013) | 0.138 0.013 0.263 0.53

Khosrowbeygi, A., et al. (2016) | -0.182 -0.990 0.627 0.28

Khosrowbeygi, A., et al. (2018) | -0.160 -0.808 0.489 0.34

Kinalski, M., et al. (2005) | 0.320 -0.170 0.810 0.40

Korkmazer, E., et al. (2015) | -0.414 -0.929 0.102 0.39

Koukkou, E., et al. (1996) | -0.930 -1.628 -0.232 0.32

Kumru, P., et al. (2016) | 0.530 0.276 0.784 0.49

Lehmann, R., et al. (2015) | -0.228 -0.479 0.024 0.50

Li, C., et al. (2013) | 0.150 -0.020 0.320 0.52

Li, D.D., et al. (2015) | 0.400 0.142 0.658 0.49

Li, G.H., et al. (2018) | 0.150 0.009 0.291 0.53

Li, G.H., et al. (2015) | 0.090 0.013 0.167 0.54

Li, H., et al. (2016) | 0.070 -0.010 0.150 0.54

Li, J., et al. (2016) | 0.700 0.396 1.004 0.48

Li, J.Y., et al. (2017) | 0.250 0.002 0.498 0.50

Li, P., et al. (2018) | -0.080 -0.253 0.093 0.52

Li, S.M, et al. (2015) | 0.280 -1.901 2.461 0.07

Li, X.M., et al (2015) | -0.335 -0.665 -0.004 0.47

Li, Y.Y., et al (2015) | 0.340 -0.017 0.697 0.46

Li. F., et al (2020) | 0.390 0.120 0.660 0.49

Liang, Z.X., et al (2016) | 1.000 0.561 1.439 0.42

Liao, Y., et al. (2018) | 2.060 0.982 3.138 0.20

Lipu, et al (1997) | 1.042 0.853 1.232 0.51

Liu, B., et al (2016) | -0.040 -0.151 0.071 0.53

Liu, B., et al (2016) | -0.070 -0.635 0.495 0.37

Liu, D., et al (2016) | 0.160 0.020 0.300 0.53

Liu, H., et al (2019) | 0.300 -0.138 0.738 0.42

Liu. L., et al (2020) | -0.168 -0.390 0.054 0.50

Liu. L., et al (2020) | -0.160 -0.453 0.133 0.48

Liu. L., et al (2020) | 0.190 0.030 0.350 0.52

Liu. L., et al (2020) | -0.150 -0.392 0.092 0.50

Liu. M., et al (2020) | 0.410 0.129 0.691 0.49

Liu. P.J. et al (2020) | -0.140 -0.344 0.064 0.51

Liu. T., et al (2020) | -0.060 -0.202 0.082 0.52

Liu. Y., et al (2021) | 0.040 -0.218 0.298 0.49

Lou, Y., et al (2014) | 0.045 -0.075 0.165 0.53

M, L., et al (2018) | 0.386 0.240 0.532 0.52

Ma, et al. (2012) | -0.265 -0.590 0.060 0.47

Maitland, R. A., et al (2014) | -0.190 -0.778 0.398 0.36

Maple-Brown, L., et al (2012) | -0.200 -0.394 -0.006 0.51

Marseille-Tremblay, C., et al (2| 0.010 -1.265 1.285 0.16

McGrowder, D., et al (2009) | 0.270 -0.546 1.086 0.28

Miettinen, H.E., et al (2014) | -0.060 -0.129 0.009 0.54

Miettinen, H.E., et al (2018) | -0.350 -0.527 -0.173 0.52

Mm, W.Q., et al (2014) | -0.228 -0.600 0.144 0.45

Molnar, J., et al (2008) | -0.100 -0.851 0.651 0.30

Montelongo, A., et al (1992) | 0.250 -0.370 0.870 0.35

Morimitsu, L.K., et al (2007) | 0.520 -0.232 1.272 0.30

Mou Y.Y., et al (2016) | -0.330 -0.569 -0.091 0.50

Mrizak, I., et al (2013) | 0.350 -0.378 1.078 0.31

Mrizak, I., et al (2014) | -0.450 -0.582 -0.318 0.53

Niu, J.M., et al (2013) | -0.200 -0.283 -0.117 0.53

Oiu, C., et al (2007) | -0.297 -0.534 -0.061 0.50

Pan, B.L., et al (2016) | 1.070 0.877 1.263 0.51

Paradisi, G., et al (2010) | 0.101 -0.488 0.690 0.36

Paradisi, G., et al (2002) | -0.186 -0.269 -0.104 0.53

Pezeshki, B., et al (2019) | 0.057 -0.089 0.203 0.52

Ping, et al. (2012) | -0.130 -0.233 -0.027 0.53

Prieto-Sanchez, M.T., et al (201| -0.199 -0.774 0.376 0.37

Qiu, Y.H., et al (2016) | -0.240 -0.358 -0.123 0.53

Ren. Z., et al (2020) | -0.110 -0.169 -0.051 0.54

Reyes-López, R., et al (2014) | 0.000 -0.257 0.257 0.49

Rizzo, M., et al (2008) | 0.100 -0.298 0.498 0.44

Roca-Rodríguez, et al. (2017) | -0.400 -1.144 0.344 0.30

Rojas, I., et al (2002) | 0.140 -0.385 0.665 0.39

Ruchat, et al. (2013) | -0.310 -0.839 0.219 0.39

Sarkar, P.D., et al (2006) | 0.085 0.020 0.150 0.54

Savona-Ventura, C., et al (2016)| -0.200 -0.361 -0.039 0.52

Savvidou, M., et al (2010) | 0.300 0.137 0.463 0.52

Shuang, W., et al (2014) | 0.100 -0.075 0.275 0.52

Siddiqui, K., et al (2018) | 1.099 0.454 1.744 0.34

Simon-Muela, I., et al (2015) | -0.260 -0.585 0.065 0.47

Sobki, S.H., et al (2004) | 0.153 -0.368 0.674 0.39

Soydinc, S., et al (2013) | -0.111 -0.502 0.279 0.44

Sreckovic, I., et al (2014) | -0.052 -1.110 1.007 0.21

Su, Y.X., et al (2010) | -0.100 -0.421 0.221 0.47

Suntio, K., et al (2010) | -0.600 -1.068 -0.132 0.41

Takhshid, M.A., et al (2015) | -0.129 -0.374 0.115 0.50

Takhshid, M.A., et al (2015) | -0.132 -0.369 0.105 0.50

Takhshid, M.A., et al (2015) | -0.233 -0.478 0.012 0.50

Tarim, E., et al (2006) | 0.054 -0.417 0.524 0.41

Tarim, E., et al (2004) | 0.155 -0.244 0.554 0.44

Todoric, J., et al (2013) | -0.359 -0.663 -0.056 0.48

Todoric, J., et al (2013) | -0.383 -0.911 0.145 0.39

Trebotic, L.K., et al (2015) | 0.858 0.131 1.584 0.31

Tsai, P.J., et al (2005) | -0.300 -0.528 -0.072 0.50

Turek, I.A., et al (2014) | 0.016 -0.295 0.327 0.47

Tuzun, D., et al (2018) | -0.209 -0.583 0.166 0.45

Tönjes, A., et al (2019) | 0.050 -0.362 0.462 0.43

Uebel, K., et al (2014) | -0.415 -1.593 0.762 0.18

Usluoğullari, B., et al. (2017) | -0.052 -0.376 0.273 0.47

Vastagh, I., et al. (2011) | 0.020 -0.390 0.430 0.43

Visiedo, F., et al (2013) | 0.357 -0.591 1.304 0.24

Vitoratos, N., et al (2002) | -0.095 -1.392 1.203 0.16

Vural, M., et al. (2012) | 0.176 -0.229 0.581 0.44

Wang, C., et al (2017) | 0.110 0.062 0.158 0.54

Wang, D.Y, et al (2013) | 0.150 -0.187 0.487 0.46

Wang, H.Y., et al (2019) | 0.095 -0.120 0.310 0.51

Wang, J., et al (2019) | 0.070 0.001 0.139 0.54

Wang, X., et al (2019) | -0.040 -0.243 0.163 0.51

Wang, Y. Y., et al (2018) | 0.210 0.043 0.377 0.52

Wang, Y.Y., et al (2019) | 0.190 -0.087 0.467 0.49

Wani. K., et al (2020) | 0.100 -0.078 0.278 0.52

Wei, J.H., et al (2014) | 1.420 1.287 1.553 0.53

Weng. Q., et al (2019) | -0.140 -0.316 0.036 0.52

White, S.L., et al (2016) | 0.000 -0.112 0.112 0.53

Whyte, K., et al (2013) | 0.120 -0.215 0.455 0.47

Wu, H., et al. (2019) | -0.200 -0.445 0.045 0.50

Wójcik, M., et al (2015) | 0.274 -0.390 0.938 0.33

Wójcik, M., et al (2014) | -0.091 -0.584 0.403 0.40

Xu, M., et al (2015) | -0.100 -0.476 0.276 0.45

Xu. H.F., et al (2020) | 0.730 0.527 0.933 0.51

Yanar, et al. (2019) | 0.300 0.127 0.473 0.52

Yang, X., et al (2017) | -0.540 -0.650 -0.430 0.53

Yang, Y., et al (2018) | 0.410 0.272 0.548 0.53

Ye, D., et al (2016) | 0.200 0.110 0.290 0.53

Yen, I.W., et al (2019) | 0.147 -0.087 0.382 0.50

Yi, J., et al (2018) | 0.090 -0.084 0.264 0.52

Yousefzadeh, G., et al (2014) | 0.083 -0.518 0.683 0.36

Yuan, T., et al (2015) | -0.250 -0.703 0.203 0.42

Yuan, T., et al (2014) | -0.200 -0.395 -0.005 0.51

Yue, C.Y., et al (2018) | -0.040 -0.215 0.135 0.52

Zakovicova , et al. (2014) | -0.400 -0.787 -0.013 0.44

Zhan, Y., et al (2015) | 0.060 -0.168 0.288 0.50

Zhang, J.W., et al (2017) | 0.250 0.144 0.356 0.53

Zhang, Y., et al (2017) | 0.100 -0.200 0.400 0.48

Zhang, Y., et al (2016) | 0.380 0.068 0.692 0.47

Zhang, Y.S., et al (2018) | 0.200 -0.158 0.558 0.46

Zhang. X.M., et al (2020) | 0.130 0.040 0.220 0.53

Zhang. Y.Z., et al. (2020) | 0.040 -0.044 0.124 0.53

Zhao, M., et al (2016) | 0.140 0.066 0.214 0.54

Zhao, M., et al (2010) | -1.210 -1.384 -1.036 0.52

Zheng, D.L., et al (2016) | -0.340 -0.553 -0.127 0.51

Zheng, R., et al (2015) | 0.450 0.348 0.552 0.53

Zheng. T., et al (2019) | 0.060 0.012 0.108 0.54

Zhong. L.Q., et al (2020) | 0.190 -0.147 0.527 0.46

Zhou, J., et al. (2018) | 0.150 -0.830 1.130 0.23

Zhou, X., et al (2017) | 0.540 0.360 0.720 0.52

Zhou, Y., et al (2016) | 0.190 0.145 0.235 0.54

Zhou. M., et al (2020) | -0.170 -0.319 -0.021 0.52

Zhu, J.P., et al (2014) | 0.090 -0.035 0.215 0.53

de Melo, S.F., et al. (2015) | 0.050 -0.157 0.257 0.51

Šimják, et al. (2018) | -0.100 -0.971 0.771 0.26

--------------------------------+----------------------------------------------

Overall, DL | 0.079 0.018 0.140 100.00

-------------------------------------------------------------------------------

Test of overall effect = 0: z = 2.531 p = 0.011
