# Supplementary material for: Association of maternal lipid profile and gestational diabetes mellitus: A systematic review and meta-analysis of 292 studies and 97,880 women
Source: eClinicalMedicine. 2021 Apr 16;34:100830. doi: 10.1016/j.eclinm.2021.100830 (PMC8102708; doi:10.1016/j.eclinm.2021.100830)
Supplement: Supplementary file 7 [file mmc7.docx]

Supplementary Table 6 Summary Weighted Mean Differences of VLDL-C from Meta-Analyses

-----------------------------------------------------------------------------

Author (Year) | Effect [95% Conf. Interval] % Weight

------------------------------+----------------------------------------------

Akturk, M.,et al (2008) | 0.220 -0.022 0.462 5.92

Altinova,A.,et al (2007) | 0.272 0.059 0.484 6.29

Bagci, H., et al (2018) | 0.122 -0.039 0.282 6.91

Bawah, A.T,, et aal (2019) | 0.552 0.447 0.658 7.48

Baykus, Y., et al. (2012) | 0.347 -0.323 1.017 2.18

Bullon,P.,et al (2014) | 0.156 0.022 0.289 7.21

Couch, S.C, et al (1998) | -0.581 -1.190 0.028 2.50

Ghafoor, S., et al. (2012) | 0.154 -0.041 0.349 6.50

Huo, Y., et al. (2015) | 0.180 -0.970 1.330 0.90

Jameshorani, M. et al. (2018) | 0.225 0.117 0.333 7.46

Li, J.Y., et al. (2017) | 0.130 0.014 0.246 7.38

Maitland, R. A., et al (2014) | 0.050 -0.552 0.652 2.54

McGrowder, D., et al (2009) | 0.010 -0.096 0.116 7.48

Montelongo, A., et al (1992) | 0.040 -0.043 0.123 7.66

Sreckovic, I., et al (2014) | 0.724 0.592 0.856 7.22

Tarim, E., et al (2006) | 0.195 0.049 0.341 7.07

Tarim, E., et al (2004) | 0.309 0.184 0.434 7.30

------------------------------+----------------------------------------------

Overall, DL | 0.216 0.100 0.332 100.00

-----------------------------------------------------------------------------

Test of overall effect = 0: z = 3.644 p = 0.000
