# Supplementary material for: Association of maternal lipid profile and gestational diabetes mellitus: A systematic review and meta-analysis of 292 studies and 97,880 women
Source: eClinicalMedicine. 2021 Apr 16;34:100830. doi: 10.1016/j.eclinm.2021.100830 (PMC8102708; doi:10.1016/j.eclinm.2021.100830)
Supplement: Supplementary file 8 [file mmc8.docx]

**Supplementary Table 7 Subgroup analysis of circulating lipid levels between women with and without gestational diabetes mellitus stratified by OGTT procedures**

| **Lipid** | **OGTT** | **No. of studies** | **WMD (mM)** | **95% CI  (mM)** | **P** | **I^2^ statistic** | **P for I^2^ statistic** | **Tau^2^** |
| --- | --- | --- | --- | --- | --- | --- | --- | --- |
| TG | 75-g, 2-h OGTT | 137 | 0.309 | 0.242~0.375 | <0.001 | 95.1% | <0.001 | 0.1295 |
|  | 100-g, 3-h OGTT | 76 | 0.459 | 0.373~0.545 | <0.001 | 90.2% | <0.001 | 0.1089 |
| TC | 75-g, 2-h OGTT | 146 | 0.114 | 0.027~0.201 | 0.010 | 95.7% | <0.001 | 0.2382 |
|  | 100-g, 3-h OGTT | 72 | 0.176 | 0.074~0.278 | 0.001 | 82.7% | <0.001 | 0.1298 |
| HDL-C | 75-g, 2-h OGTT | 128 | -0.076 | -0.107~-0.045 | <0.001 | 93.2% | <0.001 | 0.0256 |
|  | 100-g, 3-h OGTT | 70 | -0.071 | -0.107~-0.035 | <0.001 | 88.7% | <0.001 | 0.0161 |
| LDL-C | 75-g, 2-h OGTT | 131 | 0.073 | -0.012~0.157 | 0.091 | 97.0% | <0.001 | 0.2048 |
|  | 100-g, 3-h OGTT | 63 | 0.075 | -0.007~0.158 | 0.074 | 81.2% | <0.001 | 0.0677 |
| VLDL-C | 75-g, 2-h OGTT | 6 | 0.124 | 0.038~0.211 | 0.005 | 37.8% | 0.154 | 0.0039 |
|  | 100-g, 3-h OGTT | 10 | 0.206 | 0.066~0.347 | 0.004 | 86.7% | <0.001 | 0.0381 |
